# Natural Ventilation for the Prevention of Airborne Contagion

**DOI:** 10.1371/journal.pmed.0040068

**Published:** 2007-02-27

**Authors:** A. Roderick Escombe, Clarissa C Oeser, Robert H Gilman, Marcos Navincopa, Eduardo Ticona, William Pan, Carlos Martínez, Jesus Chacaltana, Richard Rodríguez, David A. J Moore, Jon S Friedland, Carlton A Evans

**Affiliations:** 1 Department of Infectious Diseases & Immunity, Imperial College London, London, United Kingdom; 2 Wellcome Trust Centre for Clinical Tropical Medicine, Imperial College London, London, United Kingdom; 3 Asociación Benéfica PRISMA, Lima, Perú; 4 Department of International Health, Johns Hopkins Bloomberg School of Public Health, Baltimore, Maryland, United States of America; 5 Hospital Nacional Dos de Mayo, Lima, Perú; 6 Hospital Nacional Daniel Carrión, Lima, Perú; 7 Hospital de Apoyo Maria Auxiliadora, Lima, Perú; University College London, United Kingdom

## Abstract

**Background:**

Institutional transmission of airborne infections such as tuberculosis (TB) is an important public health problem, especially in resource-limited settings where protective measures such as negative-pressure isolation rooms are difficult to implement. Natural ventilation may offer a low-cost alternative. Our objective was to investigate the rates, determinants, and effects of natural ventilation in health care settings.

**Methods and Findings:**

The study was carried out in eight hospitals in Lima, Peru; five were hospitals of “old-fashioned” design built pre-1950, and three of “modern” design, built 1970–1990. In these hospitals 70 naturally ventilated clinical rooms where infectious patients are likely to be encountered were studied. These included respiratory isolation rooms, TB wards, respiratory wards, general medical wards, outpatient consulting rooms, waiting rooms, and emergency departments. These rooms were compared with 12 mechanically ventilated negative-pressure respiratory isolation rooms built post-2000. Ventilation was measured using a carbon dioxide tracer gas technique in 368 experiments. Architectural and environmental variables were measured. For each experiment, infection risk was estimated for TB exposure using the Wells-Riley model of airborne infection. We found that opening windows and doors provided median ventilation of 28 air changes/hour (ACH), more than double that of mechanically ventilated negative-pressure rooms ventilated at the 12 ACH recommended for high-risk areas, and 18 times that with windows and doors closed (*p* < 0.001). Facilities built more than 50 years ago, characterised by large windows and high ceilings, had greater ventilation than modern naturally ventilated rooms (40 versus 17 ACH; *p* < 0.001). Even within the lowest quartile of wind speeds, natural ventilation exceeded mechanical (*p* < 0.001). The Wells-Riley airborne infection model predicted that in mechanically ventilated rooms 39% of susceptible individuals would become infected following 24 h of exposure to untreated TB patients of infectiousness characterised in a well-documented outbreak. This infection rate compared with 33% in modern and 11% in pre-1950 naturally ventilated facilities with windows and doors open.

**Conclusions:**

Opening windows and doors maximises natural ventilation so that the risk of airborne contagion is much lower than with costly, maintenance-requiring mechanical ventilation systems. Old-fashioned clinical areas with high ceilings and large windows provide greatest protection. Natural ventilation costs little and is maintenance free, and is particularly suited to limited-resource settings and tropical climates, where the burden of TB and institutional TB transmission is highest. In settings where respiratory isolation is difficult and climate permits, windows and doors should be opened to reduce the risk of airborne contagion.

## Abstract 2

In eight hospitals in Lima, opening windows and doors maximised natural ventilation and lowered the risk of airborne infection. Old-fashioned clinical areas with high ceilings and large windows provide greatest protection.

## Editors' Summary

**Background.:**

Tuberculosis (TB) is a major cause of ill health and death worldwide, with around one-third of the world's population infected with the bacterium that causes it *(Mycobacterium tuberculosis)*. One person with active tuberculosis can go on to infect many others; the bacterium is passed in tiny liquid droplets that are produced when someone with active disease coughs, sneezes, spits, or speaks. The risk of tuberculosis being transmitted in hospital settings is particularly high, because people with tuberculosis are often in close contact with very many other people. Currently, most guidelines recommend that the risk of transmission be controlled in certain areas where TB is more likely by making sure that the air in rooms is changed with fresh air between six and 12 times an hour. Air changes can be achieved with simple measures such as opening windows and doors, or by installing mechanical equipment that forces air changes and also keeps the air pressure in an isolation room lower than that outside it. Such “negative pressure,” mechanically ventilated systems are often used on tuberculosis wards to prevent air flowing from isolation rooms to other rooms outside, and so to prevent people on the tuberculosis ward from infecting others.

**Why Was This Study Done?:**

In many parts of the world, hospitals do not have equipment even for simple air conditioning, let alone the special equipment needed for forcing high air changes in isolation rooms and wards. Instead they rely on opening windows and doors in order to reduce the transmission of TB, and this is called natural ventilation. However, it is not clear whether these sorts of measures are adequate for controlling TB transmission. It is important to find out what sorts of systems work best at controlling TB in the real world, so that hospitals and wards can be designed appropriately, within available resources.

**What Did the Researchers Do and Find?:**

This study was based in Lima, Peru's capital city. The researchers studied a variety of rooms, including tuberculosis wards and respiratory isolation rooms, in the city's hospitals. Rooms which had only natural measures for encouraging airflow were compared with mechanically ventilated, negative pressure rooms, which were built much more recently. A comparison was also done between rooms in old hospitals that were naturally ventilated with rooms in newer hospitals that were also naturally ventilated. The researchers used a particular method to measure the number of air changes per hour within each room, and based on this they estimated the risk of a person with TB infecting others using a method called the Wells-Riley equation. The results showed that natural ventilation provided surprisingly high rates of air exchange, with an average of 28 air changes per hour. Hospitals over 50 years old, which generally had large windows and high ceilings, had the highest ventilation, with an average of 40 air changes per hour. This rate compared with 17 air changes per hour in naturally ventilated rooms in modern hospitals, which tended to have lower ceilings and smaller windows. The rooms with modern mechanical ventilation were supposed to have 12 air changes per hour but in reality this was not achieved, as the systems were not maintained properly. The Wells-Riley equation predicted that if an untreated person with tuberculosis was exposed to other people, within 24 hours this person would infect 39% of the people in the mechanically ventilated room, 33% of people in the naturally ventilated new hospital rooms, and only 11% of the people in the naturally ventilated old hospital rooms.

**What Do These Findings Mean?:**

These findings suggest that natural methods of encouraging airflow (e.g., opening doors and windows) work well and in theory could reduce the likelihood of TB being carried from one person to another. Some aspects of the design of wards in old hospitals (such as large windows and high ceilings) are also likely to achieve better airflow and reduce the risk of infection. In poor countries, where mechanical ventilation systems might be too expensive to install and maintain properly, rooms that are designed to naturally achieve good airflow might be the best choice. Another advantage of natural ventilation is that it is not restricted by cost to just high-risk areas, and can therefore be used in many different parts of the hospital, including emergency departments, outpatient departments, and waiting rooms, and it is here that many infectious patients are to be found.

**Additional Information.:**

Please access these Web sites via the online version of this summary at http://dx.doi.org/10.1371/journal.pmed.0040068.

- Information from the World Health Organization on tuberculosis, detailing global efforts to prevent the spread of TB
- The World Health Organization publishes guidelines for the prevention of TB in health care facilities in resource-limited settings
- Tuberculosis infection control in the era of expanding HIV care and treatment is discussed in an addendum to the above booklet
- The Centers for Disease Control have published guidelines for preventing the transmission of mycobacterium tuberculosis in health care settings
- Wikipedia has an entry on nosocomial infections (diseases that are spread in hospital). Wikipedia is an internet encyclopedia anyone can edit
- A *PLoS Medicine* Perspective by Peter Wilson, “Is Natural Ventilation a Useful Tool to Prevent the Airborne Spread of TB?” discusses the implications of this study

## Introduction

Infections transmitted by the airborne route are leading causes of morbidity and mortality worldwide, with tuberculosis (TB) alone causing 1.8 million deaths each year [1]. Outbreaks occur in prisons [2,3], homeless shelters [4,5], and schools [6], but it is health care facilities that may pose the greatest risk from airborne contagion by congregating infectious and susceptible individuals, resulting in frequent airborne nosocomial transmission [7–11]. This public health problem is exacerbated by HIV infection, which increases both susceptibility and hospitalisation.

In industrialised nations, optimal care for patients at risk of transmitting airborne infections includes isolation in mechanically ventilated negative-pressure rooms. Staff and visitors wear particulate respirators, and dilutional ventilation with uncontaminated air provides additional protection from disease transmission when patients generate infectious aerosols by coughing. Ventilation is usually measured in air changes per hour (ACH), with guidelines recommending 6–12 ACH for the control of TB transmission in high-risk health care settings [12]. ACH are calculated by dividing absolute room ventilation (m^3^/h) by room volume (m^3^). However, focusing on ACH alone may be misleading [13], because the absolute ventilation of a room per occupant is a major determinant of contagion in models of airborne infection, such as the Wells-Riley equation [14]. Protection against the transmission of airborne infection is increased by maximising absolute ventilation per occupant, which may be achieved by increasing the number of ACH or by increasing the room volume per occupant for a given rate of air exchange.

Dilutional ventilation with fresh air becomes critical for airborne infection control whenever infectious and susceptible people share air space without the use of particulate respirators, such as in waiting rooms, outpatient clinics, emergency departments, shared wards, and investigation suites. These spaces are often ventilated at levels well below those recommended for the control of TB transmission. Furthermore, most airborne infections such as TB occur in the developing world where isolation facilities are sparse, effective mechanical ventilation is often too costly to install or maintain, respirator use is infrequent, and wards and waiting areas are frequently overcrowded. Consequently, transmission of airborne infections to staff, relatives, and other patients is even more common in the developing world, where health care facilities may disseminate the very infections they are attempting to control.

In resource-limited settings lacking negative-pressure respiratory isolation, natural ventilation by opening windows is recommended for the control of nosocomial TB [15], but the rates and determinants of natural ventilation in health care facilities have not been defined. We therefore measured ventilation in a variety of hospital wards and clinics where infectious patients are likely to be encountered. We investigated the determinants of natural ventilation, and used mathematical modelling to evaluate the effect of natural ventilation on airborne TB transmission.

## Methods

### Setting

Ventilation was measured in 368 experiments in 70 naturally ventilated rooms in eight hospitals in Lima, Peru. These included respiratory isolation rooms (*n* = 13); wards for TB (*n* = 13), respiratory (*n* = 9), general medical (*n* = 8), and HIV/infectious diseases (*n* = 4) patients; emergency departments (*n* = 8); out-patient consulting rooms (*n* = 6); TB clinics (*n* = 5); nebuliser rooms (*n* = 2); one autopsy room; and one respiratory outpatient waiting room. Five hospitals were built pre-1950 (“old-fashioned”) and three were constructed 1970–1990 (“modern,” naturally ventilated). Lima's first mechanically ventilated negative-pressure isolation rooms for TB, built in 2000, were also studied (*n* = 12). The following architectural features and environmental variables were recorded: area of windows and doors open; presence of open windows or doors on opposite walls to facilitate the through-flow of air; ceiling height; floor area; elevation of the room above ground level; temperature; relative humidity; and wind speed measured at the window using a thermal anemometer (TA35 Airflow Technical Products, http://www.airflow.com). Direction of airflow was assessed using smoke tubes. Ethical approval was obtained from Asociación Benéfica PRISMA, Peru.

### Measurement of Ventilation

ACH were measured using a tracer gas concentration-decay technique [16]. With all windows and doors closed, carbon dioxide (CO_2_) was released and mixed well with room air using large fans to create a spatially uniform CO_2_ concentration in the room. Fans were then switched off so as not to interfere with natural ventilation air currents. Depending on room size, after 5–15 min, windows and doors were opened, either simultaneously or sequentially. CO_2_ concentrations were measured throughout at 1-min intervals using a centrally located infrared gas analyser (Gas-Data Ltd, http://www.gasdata.co.uk).

### Calculation of Air Changes per Hour

ACH were calculated for each experiment for each configuration studied: all windows/doors closed; some but not all windows/doors open; all windows/doors fully open. ACH were calculated as the gradient of the straight line through the natural logarithm of CO_2_ concentration plotted against time in hours [16]. Measurements were considered from peak concentrations after mixing (3,000–10,000 parts/million depending on room size) until concentration fell to within 200 parts/million of baseline, to allow for CO_2_ production by room occupants.

### Estimated Risk of Airborne Infection

The risk of airborne TB infection (percent of susceptible persons infected) was estimated for each ventilation experiment using a standard model of airborne infection, the Wells-Riley equation [14]: *C* = *S*(1 − *e* ^−*Iqpt*/*Q*^), where: *C* = number of new cases; *S* = number of susceptible individuals exposed; *e* = base of natural logarithms; *I* = number of infectors; *q* = number of infectious “quanta” produced per hour by infectors; *p* = pulmonary ventilation rate of susceptible individuals (0.6 m^3^/h [17]); *t* = exposure time (hours); and *Q* = absolute room ventilation (m^3^/h). A “quantum” is used to describe the “infectious dose” for TB, defined as the number of infectious particles required to cause infection in (1 − *e* ^−1^) of a susceptible population when each susceptible person breathed, on the average, one quantum of infectious particles [18]. Exposure duration was 24 h, and susceptible individuals were assumed to be unprotected by particulate respirators. To allow comparison between isolation and shared rooms, all patients in each room were assumed to have TB and produce 13 infectious quanta per hour (*q* = 13), the rate determined for an untreated TB case in a well-documented outbreak [17]. For external validity comparing natural and mechanical ventilation, all mechanically ventilated rooms were assumed to deliver the recommended 12 ACH [12], and absolute ventilation (m^3^/h) was therefore calculated by multiplying room volume (m^3^) by ACH [12].

### Statistical Analysis

All statistical analyses were performed with Stata v. 8.0 (Statacorp, http://www.stata.com) or SPSS v. 10 (http://www.spss.com). Determinants of ventilation and infection risk were first assessed by univariate regression. Three separate dependent variables were evaluated. Two were measures of ventilation. These were ACH and absolute ventilation (m^3^/h; derived by multiplying ACH by room volume). The third dependent variable was an estimate of TB transmission risk for exposure to patients producing 13 infectious quanta per hour as detailed in the preceding paragraph. The following continuous independent variables were examined: area of windows and/or doors open (m^2^); ceiling height (m); floor area (m^2^); wind speed (km/h); elevation of room above the ground (m); temperature (°C); and relative humidity (%). One categorical variable was examined: presence or absence of open windows and/or doors on opposite walls of a room. Associations with *p* < 0.15 were included in a multiple linear regression model [19]. For all regressions dependent variables were normalised by log_10_-transformation, and a generalised estimating equation [20] was used to fit clustering of observations within rooms. Modified “marginal *R*-square” values were calculated for these models [21]. The text presents median values, and graphs are “box-and-whisker plots” [22].

## Results

### Effect of Opening Windows and Doors

**Figure 1:**
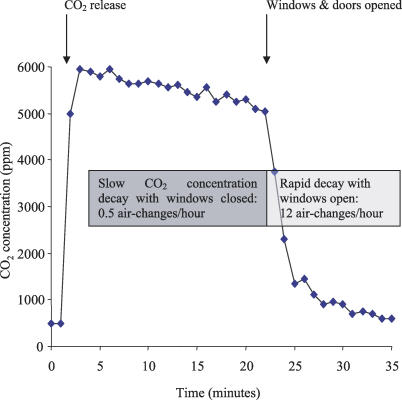
Measurement of Ventilation Illustrative carbon dioxide (CO_2_) concentration-decay experiment demonstrating a rapid rise in CO_2_ concentration during initial release to a peak of 6,000 parts/million (ppm) followed by slow decay calculated as 0.5 ACH until the windows and doors were opened. After windows and doors were opened, CO_2_ concentrations fell rapidly, indicating a calculated ventilation rate of 12 ACH. Repeated experiments of this type defined the effect of architectural and environmental variables on natural ventilation.

Changes in CO_2_ concentration were measured in each room. A characteristic pattern was observed of slow CO_2_ concentration-decay with windows and doors closed, which markedly increased on opening windows and doors. Figure 1 shows a typical concentration-decay curve, demonstrating the rapid increase in carbon dioxide removal by ventilation when windows and doors were opened. Such data was obtained for all rooms measured. For all naturally ventilated facilities, opening windows and doors provided median absolute ventilation of 2,477 m^3^/h, more than six times the 402 m^3^/h calculated for mechanically ventilated rooms at 12 ACH, and twenty times the 121 m^3^/h with windows/doors closed (all *p* < 0.001). The corresponding ACH were 28 versus 12 versus 1.5, respectively, and absolute ventilation per person was 1,053 m^3^/h versus 374 m^3^/h versus 55 m^3^/h, respectively (all *p* < 0.001).

**Figure 2:**
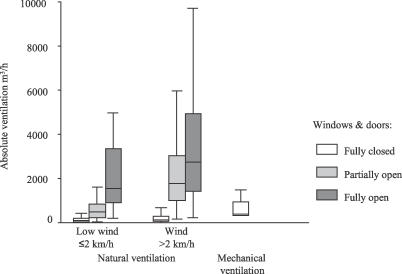
Effect of Window Opening and Wind Speed on Absolute Ventilation The effect of partial and complete window opening and wind speed on natural ventilation is shown, compared with mechanically ventilated negative-pressure respiratory isolation rooms. The triplet of bars on the left of the graph represents absolute ventilation measured in naturally ventilated clinical rooms on days when wind speed was within the lowest quartile (i.e., ≤2 km/h), with windows and doors closed (*n* = 102), partially open (*n* = 167), or fully open (*n* = 86). The triplet of bars in the centre of the graph represents absolute ventilation at wind speeds in the upper three quartiles combined (i.e., >2 km/h) with windows and doors closed (*n* = 266), partially open (*n* = 74) or fully open (*n* = 240). “Partially open” was defined as at least one window and/or door open, but not all. The single bar on the right of the graph represents absolute ventilation in mechanically ventilated negative-pressure respiratory isolation wards at 12 ACH. The corresponding median ACH for the seven bars from left to right are: 1.0; 7.6; 20; 1.8; 17; 34; and 12.

Opening increasing numbers of windows and doors increased ventilation. This is demonstrated in Figure 2 and Table 1 where absolute ventilation is shown for naturally ventilated rooms with windows and doors closed; partially open (i.e., at least one but not all of windows and doors fully open); and fully open (i.e., all windows and doors fully open). The lowest versus the upper three quartiles of wind speed combined are shown in Figure 2 and demonstrate the increase in natural ventilation with increasing wind speed and the rates of natural ventilation achieved even on relatively still days. Figure 2 also shows the absolute ventilation calculated for the 12 mechanically ventilated respiratory isolation rooms in the study, assuming they were ventilated at the 12 ACH according to guidelines for high-risk areas [12]. With windows and doors fully open even the lowest quartile of wind speeds (≤2 km/h) resulted in significantly greater ventilation than that provided by mechanical ventilation at 12 ACH (*p* < 0.001).

**Table 1:**
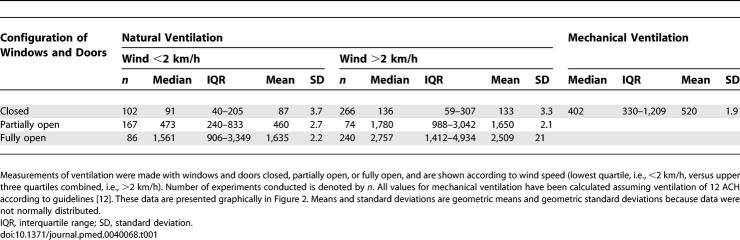
Summary Statistics for Absolute Ventilation (m^3^/h) in Naturally Ventilated Rooms compared with Mechanical Ventilation

### Old-Fashioned versus Modern Naturally Ventilated Facilities

**Figure 3:**
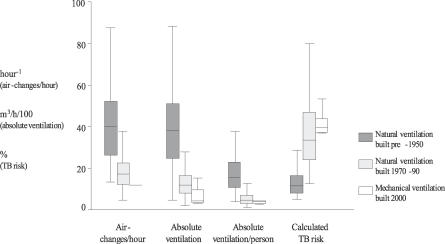
Ventilation and Protection against Airborne TB Transmission in Old-Fashioned Compared with Modern Rooms Ventilation and protection against airborne infection is shown for pre-1950 versus modern (1970–1990) naturally ventilated facilities versus mechanically ventilated negative-pressure respiratory isolation rooms. The triplet of bars on the left represents ACH in old-fashioned, high-ceilinged, pre-1950 naturally ventilated clinical areas (*n* = 22; 201 experiments), versus modern naturally ventilated facilities (*n* = 42; 125 experiments), versus mechanically ventilated negative-pressure facilities (*n* = 12). The left-centre triplet of bars represents the same comparison for absolute ventilation (m^3^/h/100); the right-centre triplet of bars represents that for absolute ventilation per person (m^3^/h/100); and the triplet of bars on the right that for the estimated risk of airborne TB transmission (percentage of susceptible persons infected), for 24-h exposure to infectious TB patients [17]. Data are shown for 64 naturally ventilated rooms with windows and doors fully open (the remaining six naturally ventilated rooms had windows that could not be fully opened).

Old-fashioned facilities built pre-1950 had greater natural ventilation than more modern rooms built 1970–1990. With windows and doors fully open, the median absolute ventilation was 3,769 versus 1,174 m^3^/hour, the median absolute ventilation per person was 1,557 m^3^/h versus 461 m^3^/h, and the ACH were 40 versus 17, respectively (all *p* < 0.001; Figure 3; Table 2). Compared with the modern naturally ventilated facilities, these pre-1950 facilities were larger (85 m^3^ versus 60 m^3^), with higher ceilings (4.2 m versus 3.0 m), larger windows (area 6.6 m^2^ versus 3.4 m^2^; window area to room volume ratio 0.1 versus 0.05) and were more likely to have windows on opposite walls allowing through-flow of air (56% versus 19% of rooms) (all *p* < 0.05). Importantly for calculations of airborne infection risk, patient crowding was similar in old-fashioned and modern wards (floor area/patient 9.2 versus 9.3 m^2^; *p* = 0.5). Floor area per patient tended to be greater in modern mechanically ventilated isolation rooms than in naturally ventilated rooms, but this difference was not significant (median floor area in mechanically ventilated rooms 11 m^2^; *p* = 0.1).

**Table 2:**
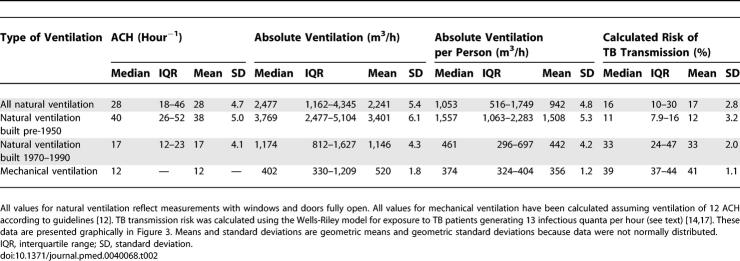
Summary Statistics for Measures of Ventilation and Calculated TB Transmission Risk

### Estimated Risk of Airborne Tuberculosis Infection

**Figure 4:**
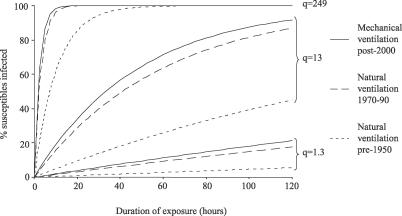
Estimated TB Transmission Risk over Time for Three Sources of Increasing Infectiousness in Naturally versus Mechanically Ventilated Facilities The estimated risk of TB infection over time for exposure to three TB source cases of different infectiousness is shown for pre-1950 naturally ventilated facilities (dotted lines) versus modern 1970–1990 naturally ventilated facilities (dashed lines) versus mechanically ventilated negative-pressure isolation facilities at 12 ACH (continuous lines). The three infectious sources are: *q* = 1.3 standard ward TB patients who infected guinea pigs studied by Riley [32] (lowest three lines); *q* = 13 an untreated TB case who infected 27 coworkers in an office over 4 wk [17] (middle three lines); and *q* = 249 for an outbreak associated with bronchoscopy of a TB patient [14] (uppermost three lines). Median values for all measures of absolute ventilation for each category of naturally ventilated room with all windows and doors open have been used in the model.

The median estimated risk of TB transmission (percentage of susceptible individuals infected) from 24 hours in rooms shared with infectious TB patients was 97% for naturally ventilated facilities with windows and doors closed, 39% in mechanically ventilated negative-pressure respiratory isolation rooms with 12 ACH of dilutional ventilation, and 33% in modern and 11% in pre-1950 naturally ventilated facilities with windows and doors fully open (Figure 3; Table 2). Figure 4 shows modelling of airborne TB transmission risk over time for pre-1950 versus modern naturally ventilated facilities versus mechanically ventilated respiratory isolation rooms at 12 ACH. Three different scenarios of increasing source infectiousness were investigated and demonstrate that the protective effect of ventilation diminishes as the infectiousness of the source increases. Figure 4 also demonstrates that the model predicts that all exposed susceptible persons eventually become infected when duration of exposure increases sufficiently.

### Determinants of Natural Ventilation

Increased natural ventilation (measured by ACH and absolute ventilation) and decreased estimated risk of TB transmission were significantly associated in multiple regression analysis with: area of windows/doors open; placement of windows/doors on opposite walls allowing through-flow of air; ceiling height; floor area; and wind speed (Table 3). Such findings were highly consistent across all three measurements (ACH, absolute ventilation, and TB transmission risk) except for ceiling height where the association with ACH was of only borderline significance (*p* = 0.056). Temperature (°C) and relative humidity (%) were also measured but did not qualify for inclusion in this model (*p* > 0.15).

**Table 3:**
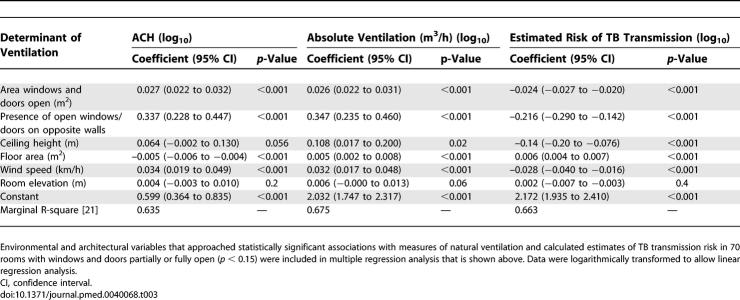
Determinants of Ventilation and Protection against Airborne TB Transmission

### Direction of Airflow

Smoke tube testing in each room demonstrated the direction of airflow through doors or windows during experiments. For 47 (67%) of the naturally ventilated rooms, in over 80% of experiments with windows and doors fully open, air currents flowed into the room through the door and passed out of the room through the window(s), or flowed into the room predominantly through one set of windows to pass out through an opposite set of windows. In 23 (33%) of the rooms, air passed into the room though the windows and out of the room through the door in over 80% of experiments with windows and doors fully open. These patterns reflected the position of a room and its windows and doors in relation to the prevailing wind in Lima.

### Mechanical Ventilation

The mechanically ventilated facility delivered less than half the number of ACH recommended when measured (unpublished data). On inspection, air extraction and supply fans were unprotected by filters, motors were poorly maintained, and fan blades were corroded and clogged with deposits. Therefore, to improve external validity, values of 12 ACH and corresponding calculated values for absolute ventilation were substituted for all comparisons between mechanical and natural ventilation.

## Discussion

We found that natural ventilation created by opening windows and doors provided high rates of air exchange, absolute ventilation, and theoretical protection against airborne TB infection. These factors were greatest in facilities built more than 50 years ago, even on days with little wind. In contrast, modern mechanically ventilated rooms had poor absolute ventilation even at recommended air exchange rates for high-risk areas, and consequently had higher estimated risks of airborne contagion.

Mechanical ventilation is expensive to install and maintain. Even in the developed world, respiratory isolation rooms often do not deliver the recommended number of ACH [23], and many fail to maintain negative pressure and may even be under positive pressure [23–25]. Such failings have been implicated in numerous TB outbreaks [7,10,26–28]. It is therefore not surprising that we found the new mechanically ventilated facility in Lima to be poorly ventilated and in need of refurbishment to achieve negative pressure and the 12 ACH recommended for the control of TB transmission in high-risk areas [12]. However, even at the recommended ventilation rate, the calculated risk of airborne contagion was greater in these mechanically ventilated rooms than in naturally ventilated rooms with open windows and doors.

Airborne infections may be prevented by screening individuals for infectiousness and isolating contagious patients in individual negative-pressure rooms in which caregivers and visitors wear particulate respirators. Respirator efficacy, however, depends on a good facial seal, which may not be easily achieved [29]. Their expense limits widespread use in resource-limited settings, and adherence to guidelines for their use is often poor, even in high-risk areas [30,31]. More importantly, respirators are rarely used when patient infectiousness is unrecognised, such as in waiting rooms and emergency departments [30], and it is these undiagnosed, untreated patients who are likely to be the most infectious [32,33]. Such patients represent an important source of nosocomial TB transmission to health care workers [23], and emergency departments may be heavily utilized by TB patients prior to diagnosis [34]. Negative-pressure isolation and dilutional mechanical ventilation are inevitably limited to selected areas that are designated high risk, such as respiratory isolation rooms. In clinical areas that are not designated high risk, including the majority of wards, emergency departments, and waiting areas, mechanical ventilation rates are usually much lower than 12 ACH, and airborne infection risks will be correspondingly higher. In the model of airborne infection with the infectious source *q* = 13 (the untreated office worker), 39% of susceptible individuals were predicted to become infected in mechanically ventilated rooms at 12 ACH, compared with 33% in modern and 11% in pre-1950 naturally ventilated facilities. If all these modern naturally ventilated hospital rooms in the study were considered instead to be mechanically ventilated at 6 ACH (a relatively high rate of ventilation for non-high-risk areas in health care settings), the model predicted that 70% of susceptible individuals would become infected. Risks of transmission would increase further were the mechanical ventilation systems to be poorly maintained. High air exchange mechanical ventilation must be reserved because of its great expense for high-risk areas. In contrast, natural ventilation is applicable across a wide variety of hospital settings, including waiting rooms, outpatient departments, and emergency departments. Indeed, it is in these areas where infectious patients are likely to be found, especially prior to diagnosis when they are untreated and therefore likely to be most infectious. Natural ventilation is also applicable in nonclinical environments such as prisons and homeless shelters, where rates of institutional TB transmission are high.

The risk of airborne contagion was significantly lower in older, spacious facilities with high ceilings and large windows on more than one wall. In contrast, modern wards with low ceilings and small windows were associated with higher risk, and mechanically ventilated rooms with sealed windows had even greater risk, despite being ventilated optimally according to guidelines. The highest risk was found in naturally ventilated rooms with all windows and doors closed, preventing almost all ventilation. Several factors may lead modern ward design to increase the risk of airborne infection. Guidelines for infection control focus on mechanical ACH rather than absolute ventilation per person. However, for a given air change rate there will be greater absolute ventilation in a larger room. For example, a 12 m^2^ isolation room with ceiling 3 m high ventilated at 12 ACH has absolute ventilation of 432 m^3^/h. The same room but with the ceiling increased to 4 m high ventilated at the same 12 ACH has absolute ventilation 576 m^3^/h and offers substantially greater protection against airborne infection according to airborne infection models. This additional protection may even be underestimated because of modelling assumptions of steady state conditions, which in reality may rarely be the case.

To prevent TB transmission, mechanical ventilation of high-risk clinical areas at a rate of 6–12 ACH is recommended [12], in part because higher rates are prohibitively expensive, noisy, and difficult to maintain. Simply opening windows and doors achieves far greater ventilation and corresponding theoretical protection against airborne infection. Probably the major reason that modern building trends increase patient risk is financial: smaller rooms (which more easily become stuffy and overcrowded) are cheaper to build and heat.

A disadvantage of natural ventilation is the difficulty in controlling direction of airflow due to the absence of negative pressure. Contamination of corridors and adjacent rooms is therefore a risk, particularly on completely still days. However, it is possible to locate a TB ward, for example, on the uppermost floor of a building and downwind of other rooms or the nursing station. Furthermore, corridors that are open at both ends may allow the passage of large volumes of fresh air that may compensate for the absence of negative pressure. The smoke pattern testing of airflow direction demonstrated consistent patterns of airflow into or out of rooms depending on the configuration of open windows and doors and location with respect to prevailing winds. In Lima prevailing winds come from the Pacific Ocean, but wind may be less predictable in other locations. The enormous dilution resulting from release of contaminated air into the outside atmosphere prevents natural ventilation from contaminating the immediate environment significantly. Whilst exhaust air from TB isolation rooms may be filtered, air from general clinical spaces is usually pumped unfiltered into the atmosphere. Consequently, opening the windows releases the same number of infectious particles into the atmosphere as mechanical ventilation without causing significant risk to those outside, but does so with greater protection for people inside the rooms.

In contrast to mechanical ventilation, natural ventilation offers high rates of air exchange for little or no cost, and is relatively free of maintenance. Whilst weather conditions play an obvious role, this study has shown that high levels of protective ventilation are readily achievable even at low wind speeds. Natural ventilation may increase building heat loss, but this may be less important in tropical climates where a large part of the burden of TB is found. Other factors such as cultural traditions or security may result in windows being tightly closed at night, but this research has demonstrated that protective rates of ventilation are achievable with windows only partially open. Furthermore, wards are less crowded during night hours, and it may also be possible to use supplementary environmental controls such as upper room ultraviolet light. Although not suited to cold regions, in temperate or tropical climates with a high prevalence of TB, it may be safer for patients, visitors, and staff to wear extra clothing in open-windowed, naturally ventilated wards and waiting rooms than to be warm in stuffy, low-ceilinged rooms with increased risk of nosocomial airborne disease transmission. Whilst this research has focused on TB transmission, natural ventilation also has implications for other infections transmitted by the respiratory route, including influenza, although it should be noted that the protective effect of ventilation diminishes as infectiousness increases [17].

There are several limitations to this study. The number of mechanically ventilated rooms included in this study (*n* = 12) was small compared with the number of naturally ventilated rooms studied (*n* = 70), which may have given an unjustly poor evaluation of mechanical ventilation in general. This possibility is mitigated by several factors. First, nine of these rooms were individual respiratory isolation rooms, and with an average volume of 31 m^3^ were typical in size. The high proportion of individual rooms in the mechanically ventilated category resulted in floor area per patient in mechanically ventilated rooms actually tending to be greater than that in naturally ventilated rooms (11 versus 9.3 m^2^ per patient), although this difference was not statistically significant. This would favour increased values for calculated absolute ventilation, and hence decreased values for transmission risk. Furthermore, mechanical ventilation was assumed to have optimal ventilation according to guidelines, 12 ACH, and it is well documented that many real-world mechanically ventilated facilities function below these recommended levels. Another limitation of the study is the inherent limitations of the Wells-Riley airborne infection model, which makes a number of assumptions such as conditions being in steady state and infection being a “one-hit” process, and does not take into account other factors such as the fact that a susceptible person located closer to an infectious source is more likely to become infected than one who is further away. The model also does not account for the deposition fraction of bacilli in the alveoli, or for the removal of viable particles from the air by processes such as settling. However, the TB transmission risk values presented are not intended as absolute estimates of risk, but rather as relative measures, to allow comparison of the protection afforded by natural ventilation in old-fashioned and modern facilities, compared with mechanical ventilation.

In summary, natural ventilation has advantages over mechanical ventilation in the fight against the institutional transmission of airborne infections, especially in resource–limited settings. When designing medical facilities there are lessons to be learnt from the past and it may be better to replace overcrowding and poor ventilation by the safer design principles of our predecessors. Well-maintained negative-pressure isolation facilities are the optimal standard of care for infectious respiratory patients. However, they are too costly for many limited-resource settings, and are restricted to small high-risk areas of health care settings, neglecting many important areas of potential transmission such as emergency departments and waiting rooms. When infectious and susceptible individuals must share rooms and respirators and negative-pressure isolation are impractical, crowding should be reduced and windows and doors opened to maximise natural ventilation and reduce the risk of airborne contagion.

## Supporting Information

Alternative Language Abstract S1 Translation of the Abstract into French by Gaeton Favre. (28 KB DOC)

Alternative Language Abstract S2 Translation of the Abstract into German by Clarissa C. Oeser. (36 KB DOC)

Alternative Language Abstract S3 Translation of the Abstract into Japanese by Mayuko Saito. (28 KB DOC)

Alternative Language Abstract S4 Translation of the Abstract into Russian by Erna Crane. (36 KB DOC)

Alternative Language Text S1 Translation of the Article into Spanish by A. Roderick Escombe and Marcos Navincopa. (344 KB DOC)

## Abbreviations

ACH: air changes per hour
TB: tuberculosis
