# Supplementary material for: Natural Ventilation for the Prevention of Airborne Contagion
Source: PLoS Med. 2007 Feb 27;4(2):e68. doi: 10.1371/journal.pmed.0040068 (PMC1808096; doi:10.1371/journal.pmed.0040068)
Supplement: Alternative Language Abstract S1 — (28 KB DOC) [file pmed.0040068.sd001.doc]

**Ventilation Naturelle pour la Prévention de la Contagion Aérogène**

Sommaire:

CONTEXTE

La transmission dans les établissements d’infections aérogènes telles que la tuberculose constitue un problème de santé publique important, particulièrement dans les établissements aux ressources limitées où des mesures de protection telles que les chambres d’isolement a pression négative sont difficiles à mettre en oeuvre. La ventilation naturelle peut constituer une autre solution peu coûteuse.

Objectifs: Etudier les débits, les déterminants et les effets de la ventilation naturelle dans les établissements de soins de santé.

METHODES ET CONSTATATIONS

Etablissements: Huit hôpitaux a Lima, Pérou, cinq hôpitaux de conception ancienne construits avant 1950 et trois de conception moderne, construits entre 1970 et 1990. 70 chambres cliniques ventilées naturellement où on est susceptible de rencontrer des patients infectieux furent étudiées. Celles-ci comprenaient des chambres d’isolement respiratoire, des salles pour patients atteints de tuberculose, des salles pour patients souffrant de maladies respiratoires, des salles de soins médicaux généraux, des cabinets de consultations externes, des salles d’attente et des services d’urgence. Ces salles furent comparées avec 12 chambres d’isolement respiratoire a pression négative ventilées mécaniquement construites après l’an 2000.

Méthodes: La ventilation fut mesurée en utilisant une technique de gaz traceur de bioxyde de carbone dans 368 expériences. Les variables architecturales et environnementales furent mesurées. Pour chaque expérience, le risque d’infection fût estimé pour l’exposition a la tuberculose en utilisant le modèle d’infection aérogène de Wells-Riley.

Constatations principales: L’ouverture des fenêtres et des portes fournissait une ventilation médiane de 28 changements d’air par heure, plus que le double de celle des chambres a pression négative ventilées mécaniquement qui sont ventilées aux 12 changements d’air par heure recommandés pour les zones a risques élevés, et dix-huit fois celle avec les fenêtres/portes fermées (p<0,001). Les installations construites il y a plus de 50 ans, caractérisées par de grandes fenêtres et de hauts plafonds, avaient une plus grande ventilation que les chambres modernes ventilées naturellement (40 par rapport a 17 changements d’air par heure; p<0,001). Même dans le quartile le plus bas de vitesses du vent, la ventilation naturelle dépassait la ventilation mécanique (p<0.001). Le modèle d’infection aérogène de Wells-Riley prédit que, dans les chambres ventilés mécaniquement, 39% de personnes susceptibles deviendraient infectées après 24 heures d’exposition a des patients atteints de tuberculose non traitée d’une infectiosité caractérisée dans une éclosion bien documentée. Par comparaison, ce pourcentage était de 33% dans les installations modernes et de 11% dans les installations ventilées naturellement datant d’avant 1950 avec les fenêtres et les portes ouvertes.

CONCLUSIONS:

L’ouverture des fenêtres et des portes maximise la ventilation naturelle de sorte que le risque de contagion aérogène est beaucoup plus bas qu’avec des systèmes de ventilation mécanique coûteux nécessitant un entretien. Les zones cliniques anciennes avec de hauts plafonds et de grandes fenêtres fournissent la protection la plus grande. La ventilation naturelle est peu coûteuse et ne nécessite aucun entretien et elle est particulièrement adaptée aux établissements a ressources limitées et aux climats tropicaux, et c’est la que la tuberculose et la transmission de la tuberculose dans les établissements sont les plus répandues. Dans les établissements où l’isolement respiratoire est difficile et où le climat le permet, les fenêtres et les portes devraient être ouvertes afin de réduire le risque de contagion aérogène.
