# Supplementary material for: Natural Ventilation for the Prevention of Airborne Contagion
Source: PLoS Med. 2007 Feb 27;4(2):e68. doi: 10.1371/journal.pmed.0040068 (PMC1808096; doi:10.1371/journal.pmed.0040068)
Supplement: Alternative Language Abstract S2 — (36 KB DOC) [file pmed.0040068.sd002.doc]

**Natuerliche Ventilation als Massnahme zur Vermeidung**

**von aerogenen Infektionen**

**Hintergrund:**

Die Uebertragung von aerogenen Infektionen wie

Tuberkulose im Bereich oeffentlicher medizinischer

Einrichtungen stellt ein schwerwiegendes Problem im

oeffentlichen Gesundheitswesen dar. Das gilt

insbesondere fuer Laender, deren finanzielle Mittel

begrenzt sind und wo Schutzmassnahmen wie die

Installierung von Isolationsraeumen, die mit

Negativdruck ausgestattet sind, aufgrund der Kosten

nur schwer moeglich sind. Natuerliche Ventilation

koennte eine Alternative sein, die mit geringen Kosten

verbunden ist.

**Zielsetzung**:

Das Ziel der Studie ist es, die Kosten, die

bestimmenden Faktoren sowie die Auswirkung der

natuerlichen Ventilation zu erforschen.

**Methoden und Erkenntnisse**

Orte der Untersuchung:

Acht Krankenhaeuser in Lima, Peru. Davon sind fuenf

Krankenhaeuser von aelterer Bauart, errichtet vor

1950. Drei Haeuser sind moderne Bauten, sie stammen

aus der Zeit zwischen 1970 und 1990. Es wurden siebzig

auf natuerliche Weise belueftete Raeume untersucht, in

denen sich vor allem Patienten mit infektioesen

Krankheiten aufhielten, d.h. Isolationsraeume auf

pulmologischen Abteilungen, TBC-Stationen,

pulmologische und allgemein internistische Stationen,

Behandlungsraeume fuer ambulante Patienten,

Warteraeume und Notfallabteilungen. Diese Raeume

wurden verglichen mit zwoelf nach 2000 erbauten,

mechanisch beluefteten Isolationsraeumen mit

Negativdruck.

**Methoden:**

Mit Hilfe von Kohlendioxyd als Indikatorgas wurde die

Ventilation bei 368 Experimenten gemessen. Ebenso

gemessen wurden architektonische und umweltbedingte

Variablen. Unter Anwendung des Wells-Riley-Modells der

aerogenen Infektion wurde bei jedem Experiment das

Infektionsrisiko, an TBC zu erkranken, bewertet.

Die wesentlichen Erkenntnisse:

Das Oeffnen von Fenstern und Tueren bewirkte eine

mediane Ventilation von 28-maligem kompletten

Raumluft-Austausch pro Stunde. Das ist mehr als das

Doppelte dessen, was in mechanisch beluefteten

Negativdruck-Raeumen geschieht, in denen ein

12-maliger Raumluft-Austausch pro Stunde fuer

Hochrisiko-Bereiche empfohlen wird. Und es ist 18-mal

mehr als das, was bei geschlossenen Fenstern und

Tueren (p<0.001) messbar ist. Einrichtungen, die vor

mehr als fuenfzig Jahren erbaut wurden, sind durch

grosse Fenster und hohe Decken gekennzeichnet. Sie

haben infolgedessen eine bessere Ventilation als

moderne, natuerlich belueftete Einrichtungen

(40-maliger gegen 17-maligen Raumluftaustausch pro

Stunde). Selbst im Bereich des niedrigsten Quartils

der Windgeschwindigkeit uebertraf die natuerliche

Ventilation die mechanische ( p<0.001). Das

Wells-Riley-Modell der aerogenen Infektion

prognostizierte, dass in mechanisch beluefteten

Raeumen 39% der Anwesenden 24 Stunden nach Kontakt mit

unbehandelten Tuberkolose-Patienten, deren

Infektiositaet aufgrund eines eindeutig dokumentierten

Krankheitsausbruch feststeht, angesteckt werden. Im

Vergleich dazu werde bei geoeffneten Fenstern und

Tueren die Anzahl der Betroffenen in modernen

Einrichtungen 33% und in natuerlich beluefteten

Raeumen aus der Zeit vor 1950 sogar nur 11% betragen.

**Schlussfolgerung**:

Das Oeffnen von Fenstern und Tueren maximiert die

natuerliche Ventilation, so dass das Risiko einer

aerogenen Ansteckung sehr viel geringer ist als bei

kostspieligen mechanischen Ventilationssystemen, die

staendiger Wartung beduerfen. Aeltere klinische

Bereiche mit hohen Decken und grossen Fenstern bieten

dabei den groessten Schutz. Natuerliche Ventilation

kostet nichts und muss nicht gewartet werden. Sie

ist daher besonders geeignet in Laendern, die ueber

wenige finanzielle Mittel verfuegen und in denen

tropisches Klima herrscht. Und gerade in diesen

Laendern ist die Belastung durch TBC und die

Uebertragung der Krankheit im Bereich medizinischer

Institutionen am hoechsten. In Institutionen, in denen

Isolation nur schwierig durchfuehrbar ist, sollten

also, wenn es die klimatischen Bedingungen erlauben,

Fenster und Tueren geoeffnet werden, um dadurch die

Gefahr aerogener Infektionen zu verringern.
