# Supplementary material for: Natural Ventilation for the Prevention of Airborne Contagion
Source: PLoS Med. 2007 Feb 27;4(2):e68. doi: 10.1371/journal.pmed.0040068 (PMC1808096; doi:10.1371/journal.pmed.0040068)
Supplement: Alternative Language Abstract S3 — (28 KB DOC) [file pmed.0040068.sd003.doc]

空気感染予防のための自然換気

要約

背景

　結核のような空気感染症の施設内感染は、陰圧の隔離室などの予防措置を講じることが難しい資源の限られた状況では、公衆衛生上特に重要な課題である。自然換気が低コストの代替手段である可能性がある。

目的：医療環境における自然換気率、規定因子、効果について調査する。

方法と結果

設定：ペルー、リマ市の8病院。1950年以前に建設された古い建築様式の５病院、1970年から90年に建てられた近代様式の3病院において、感染性疾患患者と接触する可能性が高い７０の自然換気の部屋を調査した。これらには空気感染疾患の隔離室、結核病棟、呼吸器病棟、一般内科病棟、外来診察室、待合室、救急部を含む。これらを2000年以降に建設された機械的陰圧を備えた空気感染疾患の隔離室と比較した。

方法：換気は二酸化炭素のトレーサーガスを用いた方法で368回測定された。

建築、環境因子を測定した。それぞれの実験において、感染のリスクはWells-Riley の空気感染モデルを用い結核菌暴露のリスクについて見積もった。

主な結果：　窓や扉の開放によって室内気が一時間あたり入れ替わる換気回数は28回で、これはリスクの高い環境で推奨されている12回/時を上回っており、扉や窓を閉鎖した状態の18倍であった（p<0.001）。50年以上前に建設された施設は大きな窓と高い天井を特徴とし、近代的な施設の自然換気に比べて換気に優れていた（換気回数40回 vs 17回/時、p<0.001）。風速の遅い状態の下位25％の実験においても自然換気は機械的な換気より優れていた（p<0.001）。アウトブレイク時の報告に記された未治療の結核患者の感染性をもとにWells-Riley の空気感染モデルを用いると、機械的換気による部屋では24時間以内に39％が結核に感染する可能性があると予測された。同値を窓や扉を開放した自然換気と比較すると、近代的な施設では33％、1950年以前の施設では１１％であった。

結論

　窓や扉を開放することにより自然換気は最大化し、空気感染のリスクはメインテナンスやコストがかかる機械換気システムよりも低い。高い天井と大きな窓がある旧式の診療施設の予防効果が最大であった。自然換気はほとんどコストがかからずメインテナンスも必要でないため、資源に恵まれない地域や熱帯気候の地域には適しており、かつこれらの地域は結核による疾病負担や施設内感染がもっとも高いレベルにある。空気感染症患者を隔離することが難しい状況で、気候条件が許せば、空気感染のリスクを減らすために扉や窓を開放するべきである。
