# Supplementary material for: Natural Ventilation for the Prevention of Airborne Contagion
Source: PLoS Med. 2007 Feb 27;4(2):e68. doi: 10.1371/journal.pmed.0040068 (PMC1808096; doi:10.1371/journal.pmed.0040068)
Supplement: Alternative Language Abstract S4 — (36 KB DOC) [file pmed.0040068.sd004.doc]

**Резюме:**

**Предпосылки**

Заражение инфекционными заболеваниями, передаваемыми воздушным путём в здравоохранительных учереждениях представляет серьёзную проблему для здоровья населения, особенно там, где из-за ограниченных средств, такие защитные меры как изоляторы с вытяжкой воздуха трудно внедрять.

***Цель исследования:*** Рассмотреть скорость, степень и эффективность воздухообмена (проветривания) естественным путём в здравоохранительных учереждениях.

МЕТОДЫ И ПОЛУЧЕННЫЕ ДАННЫЕ

***Учереждения:*** Были исследованы восемь больниц в Лиме, Перу. Пять больниц в зданиях старого архитектурного стиля, построенных до1950 и три нового стиля, построенных в 1970-90 годы, 70 естественно проветриваемых клинических помещений, в которых возможно присутствие заразных больных. В числе этих помещений были изоляторы с воздухообменом, туберкулёзные палаты, палаты для пациентов с заболеваниями дыхательных путей, общие палаты, кабинеты для консультирования, приёмные и отделения скорой помощи. Все эти помещения сравнивались с 12 изоляторами с механической вытяжкой, построенными после 2000 года.

***Методы:*** Вентиляция измерялась с использованием меченого углекислого газа в 368 экспериментах. Измерялись различия архитектурных стилей и условий окружающей среды. В каждом эксперименте определялся риск заражениия при контакте с туберкулёзом, используя модель Уэльс-Райли для инфекций, передаваемых воздушным путём.

***Основные данные:*** При открытых окнах и дверях средний воздухообмен в час составлял 28, что больше чем в два раза превышал воздухообмен в помещениях с механической вытяжкой, где он составлял 12, что рекомендуется для помещений с высокой опасностью заражения и в 18 раз выше, чем при закрытых окнах/дверях (р<0.001). Здания, построенные >50 лет назад с большими окнами и высокими потолками обеспечивают больший естественный воздухообмен, чем современные (40 против 17 воздухообмен/час; р<0.001). Даже при наименьшей скорости ветра (взятой как четверть всех наименьших скоростей) естественный воздухообмен выше, чем в помещениях с механической вытяжкой (р<0,001). Модель Уэльс-Райли для инфекций передаваемых воздушным путём, предполагает, что в помещениях с механической вытяжкой 39% восприимчивых заразятся после 24 часового контакта с туберкулёзными больными, не проходящими курс лечения, как свидетельствуют документы вспышки эпидемии, по сравнению с 33% в современных зданиях и 11% в зданиях, построенных до 1950 года в помещениях с естественным воздухообменом при открытых окнах и дверях.

**ВЫВОДЫ.**

Открытые окна и двери обеспечивают максимальный естественный воздухообмен значительно уменьшая риск заражения заболеваниями, передаваемыми воздушным путём, по сравнению с риском в помещениях с механическими вытяжками. Клинические помещения в старых зданиях с большими окнами и высокими потолками обеспечивают наибольшую защиту от заражения. Естественный воздухообмен, проветривание, стоит не дорого и не требует затрат на эксплуатацию, что особенно важно там, где нет достаточных средств и в тропиках, именно там, где высоки туберкулёзные заболевания и возможность заражения ими в учереждениях. Там, где трудно обеспечить изоляцию больных и где климат позволяет, окна и двери надо держать открытыми для уменьшения риска заражения заболеваниями, передаваемыми воздушным путём.

**Естественное проветривание помещений для предотвращения заболеваний передаваемых воздушным путём.**
