# Supplementary material for: Natural Ventilation for the Prevention of Airborne Contagion
Source: PLoS Med. 2007 Feb 27;4(2):e68. doi: 10.1371/journal.pmed.0040068 (PMC1808096; doi:10.1371/journal.pmed.0040068)
Supplement: Alternative Language Text S1 — (344 KB DOC) [file pmed.0040068.sd005.doc]

La ventilación natural para prevenir la transmisión de enfermedades por vías respiratorias.

# Autores:

A Roderick Escombe^1,2^, Clarissa C Oeser^2^, Robert H Gilman^2,3^, Marcos Navincopa^4^, Eduardo Ticona^4^, William Pan^3^, Carlos Martínez^4^, Jesus Chacaltana^5^, Richard Rodríguez^6^, David AJ Moore^1,2^, Jon S Friedland^1^, Carlton A Evans^1,2,3^.

1. Department of Infectious Diseases & Immunity y Wellcome Trust Centre for Clinical Tropical Medicine, Imperial College London, UK

2. Asociación Benéfica PRISMA, Carlos Gonzales 251, Urb. Maranga, Lima, Perú

3. Johns Hopkins Bloomberg School of Public Health, Baltimore, USA

4. Hospital Nacional Dos de Mayo, Lima, Perú

5. Hospital Nacional Daniel Carrión, Avenida Guardia Chalaca, Lima, Perú

6. Hospital de Apoyo Maria Auxiliadora, Lima, Perú

**Corespondencia a:** Dr Rod Escombe, Department of Infectious Diseases & Immunity, Imperial College London, Hammersmith Hospital campus, Commonwealth Building, Du Cane Road, London, W12 ONN, UK. [rod.escombe@imperial.ac.uk](mailto:rod.escombe@imperial.ac.uk), Telefono +511 464 0221 Fax: +1 410 510 1284

**Titulo corto:** Ventilación natural

**Numero de palabras:** *Abstract*: 377 *Articulo principal*: 3,749

**Abstract:**

***BACKGROUND***

La transmisión de enfermedades por vias respiratorias es problema grave en hospitales, especialmente en países de escasos recursos donde las medidas de protección como salas de aislamiento con presión negativa son difíciles lograr. La ventilación natural podría ofrecer una alternativa de bajo costo. Nuestro objectivo fue investigar las tazas, determinantes, y efectos de la ventilación natural en establecimientos de salud.

***METODOS y RESULTADOS***

El estudio se realizó en 8 hospitales de Lima, Peru. 5 hospitales de diseño antiguo, construido pre-1950, y 3 de diseño moderno, construido 1970-90. En estos hospitales se estudiaron 70 salas con ventilación natural donde se podría encontrar pacientes infectados, incluyendo salas de aislamiento respiratorio, pabellones para pacientes con TBC, pabellones de medicina interna, consultorios externos, salas de espera y servicios de emergencia. Estas salas fueron comparadas con 12 salas de aislamiento respiratorio con ventilación mecánica y presion negativa construidas post-2000. Ventilación fue medida usando un método de caída de gas carbónico en 368 experimentos. Diferencias arquitectonicas y ambientales fueron medidos. Por cada experimento, se estimó el riesgo de infección con tuberculosis usando el Wells-Riley modelo matemático de infeccion por el aire.

Abriendo ventanas y puertas resultó una mediana de 28 recambios-de-aire/hora, mas de doble de las salas con ventilación mecánica, ventilados a los 12 recambios-de-aire/hora recomendados para zonas de alto riesgo, y 18 veces mas que las salas con ventanas/puertas cerradas (p<0.001). Ambientes construidos hace >50 años, caracterizados por ventanas grandes y techos altos, tenían mas ventilación que salas modernas con ventilación natural (40 *vs.* 17 recambios-de-aire/hora; p<0.001). Incluso dentro del quartil mas bajo de velocidad de viento, ventilación natural excedió la mecánica (p<0.001). Según el modelo de infección por el aire Wells-Riley, el riesgo de infección fue 39% en salas con ventilación mecánica por exposición de 24 horas a pacientes tuberculosos sin tratamiento de infectividad documentado en un brote en la literatura. Este risego se compara con 33% en modernas y 11% en salas pre-1950 con ventilación natural con todas las ventanas y puertas abiertas.

*CONCLUSIONES*

La ventilación natural es maximizado por abrir puertas y ventanas, siendo el riesgo de transmisión de enfermedades por vias respiratorias mucho menor que la ventilación mecánica, que es costoso y requiere mantenimiento. Las salas de diseño antiguo con techos altos y ventanas grandes dan más protección. La ventilación natural cuesta poco y no requiere de mantenimiento, y es especialmente apta para lugares de escasos recursos y climas tropicales, y son estos mismos lugares donde se encuentra la mayor carga de TBC y transmisión institucional de TBC. En situaciones donde aislamiento respiratorio es difícil y el clima lo permite, se recomienda abrir las puertas y ventanas para poder disminuir el riesgo de transmisión de enfermedades por vias respiratorias.

Introducción:

Infecciones transmitidas por vías respiratorias son causas de morbilidad y mortalidad importante a nivel mundial, solo la tuberculosis causa 1.8 millón muertos/año[1]. Brotes suceden en penales[2, 3], albergues[4, 5] y colegios[6], pero son los establecimientos de salud que pueden generar el riesgo mas alto de transmisión de enfermedades por el aire por juntar gente infecciosa y susceptible en el mismo lugar, que resulta en transmisión nosocomial frecuente[7-11]. Este problema de salud pública ha empeorado debido al VIH, que aumenta la susceptibilidad del infectado e incrementa el número de sus hospitalizaciónes.

En países industrializados, el mejor manejo para pacientes en riesgo de transmitir enfermedades por vías espiratorias incluye el aislamiento en salas con ventilación mecánica y presión negativa. Trabajadores y visitantes llevan respiradores, y tambien hay protección de transmisión por dilución de la concentración de partículas en el aire por medidas de ventilación con aire fresco. Esto normalmente se mide como recambios-de-aire por hora. Las pautas recomiendan 6-12 re-cambios de aire por hora para el control de la transmisión de tuberculosis en zonas de alto riesgo en establecimientos de salud[12]. Se calculan re-cambios de aire por hora al dividir la ventilación total de una sala (m^3^/h) por el volumen de la sala (m^3^). Pero enfocar solo en re-cambios de aire por hora pueda ser engañoso, [13] porque es en verdad la ventilación absoluta (total) de una sala por persona es un determinante mas importante de contagio en modelos de la transmisión de enfermedades por vías respiratorias, como el modelo de Wells-Riley [14]. Protección contra la transmisión de enfermedades por el aire se logra con medidas como aumentar la ventilación total por persona, que se puede lograr por aumentar la cantidad de recambios de aire por hora, o también por una cantidad idéntica de recambios de aire, aumentar el volumen de sala por persona.

La ventilación diluciónal será crítica para el control de enfermedades transmitidas por vías respiratorias siempre y cuando pacientes infecciosos y gente susceptible compartan el mismo espacio de aire sin usar respiradores. Esto sucede en salas de espera, consultorios externos, salas de emergencia, pabellones con múltiples oocupantes, corredores y unidades de investigación por ejemplo. En hospitales que cuentan con ventilación mecánica, estos ambientes mencionados arriba frecuentemente están ventilados con niveles de recambios de aire mucho más bajo que los niveles recomendados para el control de transmisión de la tuberculosis. Y más importante, la mayoría de tuberculosis se encuentra en países de escasos recursos, donde las salas de aislamiento respiratorio son escasas, y donde sistemas de ventilación mecánica efectiva son demasiado costosos tanto en su instalación y mantenimiento. También, el uso de respiradores es menos frecuente, y los pabellones a menudo están sobre poblados de pacientes. En consecuencia, la transmisión de la tuberculosis hacia los trabajadores de salud, visitas y otros pacientes es sumamente alto en paises de escasos recursos y los establecimientos de salud podrían diseminar las mismas enfermedades las cuales están tratando de controlar.

En países de escasos recursos que no cuentan con salas de aislamiento respiratorio a presión negativa y ventilación mecánica, la ventilación natural con solo abrir las ventanas esta recomendado para el control de la transmisión intrahospitalaria de la tuberculosis.[15] Pero las tazas y determinantes de la ventilación natural en establecimientos de salud nunca han sido definidos. Entonces medimos la ventilación en varios pabellones de hospitalización y clínicas donde se puede encontrar pacientes infecciosos. Investigamos los determinantes de la ventilación natural, y usamos un modelo matemático para poder evaluar el efecto de la ventilación sobre la transmisión de la tuberculosis intrahospitalaria.

**Métodos:**

***Ubicación:*** Se midio la ventilación con 368 experimentos en 70 salas con ventilación natural en ocho hospitales en Lima, Perú. Se incluyeron salas de aislamiento respiratoria (n=13); pabellones para tuberculosis (n=13) pabellones para neumología (n=9) medicina interna (n=8) y enfermedades infecciosas/VIH (n=4); servicios de emergencia (n=8); consultorios externos (n=6); salas de los Programas de Control de TBC (n=5); salas de nebulizacion (n=2); una sala de necropsia; y una sala de espera para consultorios externos de neumología. Cinco hospitales fueron construidos antes de 1950, y tres fueron construidos entre 1970-90. Las primeras salas en el Perú con ventilación mecánica y presión negativa para pacientes con tuberculosis y VIH, construidas en 2000, fueron estudiados tambien (n=12). Los siguientes detalles arquitectónicos y variables ambientales fueron notados: área de ventanas y puertas abiertas; presencia de ventanas o puertas abiertas por paredes opuestas de una sala para poder dejar pasar corrientes de aire; altura del techo; área del piso; elevación sobre la tierra; temperatura; humedad relativa; y la velocidad del viento fue medido en las ventanas usando un anemómetro termal (TA35 Airflow Technical Products Inc, Andover, USA). La dirección del aire fue medido usando tubos de humo. Aprobación etica fue obtenido desde Asociación Benefica PRISMA, Peru.

***Cuantificación de ventilación:*** Recambios de aire por hora fueron medidos a través de una técnica de caída de concentración de tracer gas [16]. Se echo gas carbónico en una sala con todas las ventanas y puertas cerradas. Se mezclo bien el aire con ventiladores de mesa para poder crear una concentración uniforme de CO_2_ en la sala. Después se apagaron los ventiladores para no interferir con las corrientes de ventilación natural. Dependiendo del tamaño de la sala, después de 5-15 minutos, se abrieron las ventanas y puertas, simultáneamente, o en secuencia. Se midieron las concentraciones de gas carbónico cada minuto usando un medidor infra-rojo de gas ubicado en el centro de la sala (Gas Data Ltd, Coventry, UK).

***Cálculo de recambios de aire por hora (RAH):*** Se calcularon recambios de aire por hora por cada experimento por cada situación medida en los ambientes: todo cerrado (todas las puertas y ventanas cerradas); parcialmente abierta (algunas pero no todas de las puertas o ventanas abiertas); ‘todo abierto’ (todas las puertas y ventanas abiertas). Se calcularon los recambios de aire por hora, midiendo la inclinacion de la línea recta dibujado por el grafico del logaritmo natural de la concentración de gas carbónico con el tiempo en horas[16]. Se consideraron mediciones desde el pico después del intercambio de gases (3,000-10,000 partes/millón dependiendo el tamaño de la sala) hasta que disminuyó la concentración de gas carbónico dentro de 200 partes/millón del nivel basal, para tener en cuenta la cantidad de gas carbónico producido por las personas presentes en las salas.

***Riesgo de transmisión de tuberculosis por el aire:*** El riesgo de transmisión de tuberculosis por el aire (porcentaje de personas susceptibles infectados) fue estimado por cada experimento usando un modelo estándar de infeccion por el aire, la ecuación Wells-Riley[14]: C = S (1-e^-Iqpt/Q^) donde C = numero de casos nuevos, S = el numero de personas susceptibles expuestos, e = la base de logarítmicos naturales, I = el numero de pacientes infecciosos, q = la infectividad de cada infectante, en ‘quanta producida por hora’, p = ventilación pulmonar de los personas susceptibles (m^3^/h) (0.6 m^3^/hour [17]), t = tiempo de exposición (horas) y Q = la ventilación total de la sala (m^3^/h). Un ‘quantum’ es la cantidad de núcleos de la gotita de tuberculosis suficiente para producir infección, una vez inhalado.[18] Se asumió que la duración de exposición fue 24 horas, y los susceptibles no llevaron respiradores. Para poder comparar las salas de aislamiento con salas de pacientes múltiples, se asumió que todos los pacientes estuvieron infectados con tuberculosis, produciendo 13 ‘quanta’ por hora, como se reportó en un brote de un paciente antes de su tratamiento bien documentado[17]. Para la validación externa en la comparación de ventilación natural y mecanica, se asumió que todas las salas con ventilación mecánica tuviera los 12 recambios de aire por hora recomendados [12], entonces la ventilación total (m^3^/h) fue calculado multiplicando el volumen de la sala (m^3^) por recambios de aire por hora (12).

***Análisis estadístico:*** (Stata versión 8.0, Statacorp LP, Texas y SPSS versión 10, SPSS Inc, Chicago, USA) Los determinantes de ventilación y riesgo de infección fueron evualuados inicialmente por regresión univariado. Se evaluaron tres variables dependientes, de los cuales dos mediciones fueron de ventilación: recambios de aire por hora; y ventilación total (m^3^/h; derivado de multiplicar recambios de aire por hora por volumen de la sala). La tercera variable dependiente fue una estimacion de riesgo de transmisión de TB, por exposición a pacientes produciendo 13 ‘quanta infecciosa’ por hora como se mencionó arriba. Las siguientes variables independientes continuas fueron estudiadas: área de ventanas y puertas abiertas (m^2^); altura del techo (m); área del piso (m^2^); la velocidad del viento (km/h); elevación sobre la tierra (m); temperatura (^0^C); y humedad relativa (%). Una variable categórica fue examinado: presencia o ausencia de ventanas o puertas abiertas entre paredes opuestas de una sala. Asociaciones con p<0.15 fueron incluidos en un modelo de regresión múltiple lineal[19]. Para todas las regresiones, variables dependientes fueron normalizados por transformación logarítmica (base 10) y se usaron un ecuación de estimación generalizada[20] para calcular el efecto de agrupamiento de mayor observaciones hechas en algunas salas. Valores ‘R-cuadrado marginales’ modificados fueron calculados por estos modelos[21]. En el texto se presenta valores medianos, y los gráficos son ‘box-and-whisker plots’.[22]

# Resultados:

***Efecto de abrir ventanas y puertas:*** Cambios en la concentración de CO2 fueron medidos en cada ambiente. Un patrón característico de cambios en la concentración de gas carbónico fue observado: caída lenta de concentración con puertas y ventanas cerradas, y caída rápida con puertas y ventanas abiertas. Figura 1 demuestra una curva de caída de concentración de gas carbónico muy típico, con un incremento rápido en la desaparición de gas CO_2_ debido a la ventilación cuando se abrieron las puertas y ventanas. Gráficos parecidos fueron obtenidos para todas las salas donde se hizo mediciones. Para todos los ambientes ventilados de manera natural, abriendo puertas y ventanas y resultó en una mediana de ventilación total de 2,477 m^3^/h, mas de seis veces de la 402 m^3^/h calculado pora salas con ventilación mecánica y 20 veces mas de la 121 m^3^/h en las salas con ventilación natural con todo cerrado (p<0.001). Los valores correspondientes para recambios de aire por hora fueron 28 *vs*. 12 *vs*. 1.5 respectivamente, y por ventilación absoluta por persona fueron 1053 m^3^/h *vs*. 374 m^3^/h *vs*. 55 m^3^/h respectivamente.

Abriendo más ventanas aumentó la ventilación. Esto se ve en la Figura 2 y Tabla 1 donde se muestra la ventilación absoluta para salas con ventilación natural con todas las puertas y ventanas cerradas (todo cerrado); parcialmente abierta (a lo menos una pero no todas las ventanas abiertas) o todas las ventanas y puertas abiertas (todo abierto). En la figura 2 se puede observar también el quartil mas bajo de velocidades de vientos *vs*. los tres quartiles de velocidades más fuertes combinados para poder mostrar el efecto del viento en la ventilación natural. El gráfico también muestra la ventilación derivada de asumir unos 12 recambios de aire por hora según las pautas[12] en las salas de aislamiento respiratorio con ventilación mecánica. Incluso en días con poco viento (≤2 km/hora), la ventilación natural con todas las puertas y ventanas abiertas producía una ventilación significativamente mayor que la de las salas con ventilación mecánica (p<0.001).

***La ventilación natural en salas de diseño antiguo vs. Salas de diseño moderno:*** Salas de diseño antiguo construidas antes de 1950 tenian mayor ventilación natural que las salas modernas construidas entre los años 1970-1990. Con todas las ventanas y puertas abiertas, la mediana de ventilación absoluta fue 3,769 *vs.* 1,174 m^3^/h, la mediana de ventilación absoluta por persona fue 1557 m^3^/h *vs*. 461 m^3^/h, y los recambios de aire por hora fue 40 *vs.* 17 respectativemente (todo p<0.001; Tabla 2 y figura 3). En comparación con salas modernas ventiladas naturalmente, estas salas antiguas fueron mas grandes (volumen 85 *vs*. 60 m^3^), con techos mas altos (4.2 m *vs.* 3.0 m), ventanas mas grandes (área 6.6 *vs.* 3.4 m^2^; ratio de área de ventanas/ volumen de sala 0.1 *vs.* 0.05) y tenían mas probabilidad de tener ventanas ubicadas por paredes opuestas para dejar pasar corrientes de aire (56% *vs.* 19% de las salas) (todo p<0.05). Importante para los cálculos de riesgo de infección por el aire, el hacinamiento de pacientes fue similar en las salas construidas antes de 1950 en comparación con salas modernas (área del piso/paciente 9.2 *vs.* 9.3 m^2^; p=0.5). Área del piso por paciente tenia tendencia ser un poco mayor en salas con aislamiento respiratorio con ventilación mecánica pero esta diferencia no fue estadísticamente significativa (median 11 m^2^; p=0.1).

***Riesgo estimado de infección por el aire con tuberculosis:*** El riesgo mediano de transmisión de tuberculosis (porcentaje de personas susceptibles infectados) al estar 24 horas en las salas con pacientes con tuberculosis fue 97% en salas con ventilación natural con todas las puertas y ventanas cerradas, 39% en salas de aislamiento respiratorio con ventilación mecánica y presión negativa y 12 recambios de aire por hora, 33% en salas con ventilación natural construidas 1970-90, y 11% en salas antiguas construidas antes de 1950 con ventilación natural con todas las puertas y ventanas abiertas (Tabla 2 y figura 3). Figura 4 muestra mas modelos matemáticos de riesgo de infección por tiempo en tres escenarios con diferentes fuentes de infecciosidad, para las salas antiguas y modernas con ventilación natural, y las salas de aislamiento con 12 recambios de aire por hora de ventilación mecánica. Se ve como que a más tiempo todos los susceptibles se convertirán en infectados, y que la protección relativa de la ventilación es progresivamente menor cuando la infeciosidad de la fuente infectante aumenta.

***Determinantes de la ventilación natural:*** Mayor ventilación natural (medido por recambios de aire por hora y ventilación absoluta (m^3^/h) y menor riesgo de transmisión de la tuberculosis fueron asociados en regresión múltiple con: área de ventanas o puertas abiertas; ubicación de ventanas o puertas abiertas por paredes opuestas, para dejar pasar corrientes de aire; altura del techo; área del piso; y velocidad del viento (Tabla 3). Se midieron temperatura (°C) y humedad relativa (%) pero no fueron significativos para incluir en el modelo (p>0.15).

*Rumbo de corrientes de aire:* Pruebas con tubos de humo en cada sala demostro la dirección de corrientes de aire por puertas y ventanas durante los experimentos. En 47 (67%) salas con ventilación natural, en mas de 80% de los experimentos con todas las puertas y ventanas abiertas, corrientes de aire entraban a la sala por la puerta y salian por las ventanas, o entraban a la sala mayormente por un grupo de ventanas y salian por un grupo de ventanas del lado opuesto. En 23 (33%) salas, el aire entraba a la sala por las ventanas y salia por la puerta en >80% de los experimentos con ventanas y puertas completamente abiertas. Estos patrones reflejan la ubicación de la sala, sus puertas y sus ventanas en relacion al viento prevalente de Lima.

***Ventilación mecánica:*** Las salas de aislamiento con ventilación mecánica proporcionaron al medir menos de la mitad de los numeros de recambios de aire por hora recomendados (datos no publicados). Por inspección de los ventiladores de extracción y suministro de aire carecían de proteccion por filtros, falta de mantenimiento a los motores, y las aletas de los extractores estaban oxidadas y saturadas con suciedad. Entonces, para mejorar la validez externa del estudio, valores de 12 recambios de aire por hora y los valores calculados correspondientes por la ventilación total fueron usados para todas las comparaciones entre ventilación mecanica y natural.

Discusión:

Ventilación natural a través de abrir puertas y ventanas provee altas tazas de recambios de aire, ventilación total, y teóricamente protección contra la transmisión de la tuberculosis por el aire. Esta ventilación y protección fue mayor en salas construidas más de 50 años atrás, aún en días con poco viento. En contraste, salas modernas con ventilación mecánica tenía menor ventilación absoluta, a pesar de ser ventiladas a 12 recambios de aire por hora según las pautas, y tenían riezgos mas altos de transmisión de tuberculosis por el aire.

La ventilación mecánica es costosa para instalarlo y mantenerlo. Aun en países desarrollados, salas de aislamiento respiratorio con ventilación mecánica frecuentemente no dan los recambios de aire recomendados[23], y muchos fracasan en mantener presión negativa permanente[23-25]. Estas fallas han sido implicados en numerosos brotes[7, 10, 26-28]. Entonces no fue una sorpresa encontrar las nuevas salas con ventilación mecánica en Lima con poca ventilación y necesitando una renovación para lograr presión negativa y los 12 recambios de aire por hora recomendados para el control de transmisión de TBC en áreas de alto riesgo[12]. Sin embargo, aun con este nivel de ventilación recomendado, el riesgo calculado de transmisión de tuberculosis fue mas alto en estas salas con ventilación mecánica que en las salas con ventilación natural con sus puertas y ventanas abiertas.

Es posible prevenir las infecciones transmitidas por vías respiratorias a través de escreening de pacientes potencialmente más infecciosos, aislando a estos pacientes en salas de aislamiento con presión negativa, haciendo que los trabajadores y visitas usen mascarillas respiradoras. Sin embargo, la eficacia de los respiradores depende de una buena adherencia con la cara que es difícil lograrlo[29]. El costo de respiradores limita el uso de estos en países de escasos recursos, y a pesar de las normas que recomiendan utilizarlas la costumbre para su uso es bajo en muchas instancias aun en zonas de alto riesgo[30, 31]. Algo importante, raras veces se usan respiradores cuando la infecciosidad de pacientes aparentemente no esta reconocido, es decir antes del diagnóstico, por ejemplo en salas de espera, consultorios externos y salas de emergencia[30], y son estos pacientes, sin diagnóstico todavía, y sin tratamiento todavía, los cuales pueden ser mas infecciosas.[32, 33] Este tipo de pacientes representa una fuente importante de la transmisión de la TBC nosocomial para trabajadores de salud [23], y los servicios de emergencia podrian ser usados mucho por pacientes tuberculosos antes de su diagnóstico[34]. Además, la ventilación mecánica a presión negativa es por su costo es obviamente limitada a zonas específicas, designadas como alto riesgo (por ejemplos salas de aislamiento respiratorio). En zonas no designadas de alto riesgo, que incluye la mayoria de pabellones, servicios de emergencia, y salas de espera, tazas de recambios de aire por hora de ventilación mecanica suelen ser mucho menor que los 12 recambios de aire por hora, y el riesgo de transmisión de enfermedades por el aire correspondientemente es mas alto. En el modelo matematico, usando la fuente de infección q=13 (la trabajadora en una oficina con TB sin tratamiento), 39% de personas susceptibles se previeron ser infectados en salas con ventilación mecanica de 12 recambios de aire por hora, en comparación con 33% en salas modernas y 11% en salas pre-1950 con ventilcion natural. Si todas estas salas modernas con ventilación natural en el estudio fueran en cambio consideradas como con ventilación mecanica de 6 recambios de aire por hora (un nivel relativamente alto de ventilación para zonas no designados alto riesgo en establecimientos de salud), el modelo previó que 70% de personas susceptibles se contagiarian. Estos riesgos de infeccion subirian si los sistemas de ventilación mecanica no recibieran buen mmantenimiento. En contraste con la ventilación mecanica que es reservado para zonas de alto riesgo, la ventilación natural es aplicable en una gran variedad de ambientes en establecimientos de salud, incluso en emergencias, consultorios externos, y salas de espera. Es en estas zonas donde se suelen encontrar a los pacientes infecciosos, especialmente antes de su diagnostico y sin tratamiento. La ventilación natural también es aplicable en lugares fuera de los hospitales donde hay alto riesgo de transmisión de la tuberculosis, como en penales o albergues, lugares donde se encuentran altos niveles de transmisión de tuberculosis.

El riesgo de la transmisión de la tuberculosis fue mucho menor en salas de diseño antiguo, con techos altos y ventanas grandes y con ventanas en paredes opuestas. En contraste, salas modernas, con techos bajos y ventanas pequeñas fueron asociados con un riesgo de transmisión de tuberculosis más alto, y las salas con ventilación mecánica tenían un riesgo más alto todavía a pesar de ser ventilado según pautas. El riesgo más alto de todas fue en salas con todas las puertas y ventanas cerradas que impide todo tipo de ventilación. Varios factores hacen que el diseño moderno de salas de hospitalización aumenta el riesgo de la transmisión de la tuberculosis. Las pautas enfocan en recambios de aire por hora en vez de ventilación absoluta por persona. Sin embargo, por un valor fijo de recambios de aire por hora, será más ventilación absoluta en una sala que es más grande. Por ejemplo, una sala de aislamiento con área de piso de 12 m^2^ y techo de 3 m de altura ventilada a 12 recambios de aire por hora tiene una ventilación absoluta de 432 m^3^/h. La misma sala pero con techo de 4 m ventilado a 12 recambios de aire por hora tiene una ventilación absoluta de 576 m^3^/h, y ofrece mucho mas protección contra infecciones por vías respiratorias según los modelos matematicos de infeccion aerea. Esta proteccion adicional aun podria ser una sub-estimacion porque los modelos asumen condiciones de estado equilibrado las cuales en realidad pocas veces existen.

Para prevenir la tuberculosis se recomiendan 6-12 recambios de aire por hora [12] para la ventilación mecánica de áreas de alto riesgo en parte porque tazas de ventilación mas altas son prohibitivamente costosas, ruidosas por vibración de motores y ductos, y difíciles para mantenerlos. Simplemente abriendo las ventanas y puertas logra una ventilación sumamente mas alta y proteccion paralela teórica contra la infeccion aerea. Probablemente la mayor razón en la construccione de hospitales modernos y que aumenta el riesgo de transmisión de la tuberculosis es debido al ahorro en los costos: salas pequeñas y hacinadas y mal ventiladas son más baratos para construir, y para mantenerlas calidas.

Un riesgo de la ventilación natural es que no hay control del rumbo del aire contaminado debido a la ausencia de presion negativa. La contaminación aerea de corredores y salas acostadas es un riesgo entonces, especialmente en dias con poco viento. Pero seria posible, por ejemplo, ubicar un pabellón para TBC en el piso mas alto de un edificio, y ‘downwind’ de las otras salas y la estacion de enfermeras. Ademas, corredores abiertos por ambos ende(extremos) podrian dejar pasar volúmenes muy grandes de aire fresco que podria compensar la ausencia de presion negativa. Las pruebas de rumbo de aire con tubos de humo mostraron patrones consistentes de corrientes de aire hacia adentro o hacia fuera de las salas, dependiendo de la configuración de puertas y ventanas abiertas, y la ubicación de la sala con respecto a los vientos prevalentes. En Lima, los vientos prevalentes vienen desde el océano pacifico, pero el rumbo del viento podria ser menos predicible en otras locaciones. Pero al salir hacia afuera, la dilución de partículas en el aire contaminado es casi infinita, por lo que el riesgo para el ambiente de alrededor sea mínimo con la ventilación natural. Mientras que el aire de escape de salas de aislamiento respiratorio para TBC podria ser filtrado, aire desde otras zonas suelen ser expulsado al medio ambiente sin filtración. En consecuencia, al abrir las ventanas deja escapar la misma cantidad de particulas infecciosas hacia el atmosfera como la ventilación mecanica, sin causar riesgo significativo a los que esten afuera, pero con proteccion mayor para la gente dentro de las salas.

En contraste con la ventilación mecánica, la ventilación natural ofrece altos niveles de recambios de aire por poco costo, y no requiere de mayor mantenimiento. El clima tiene su papel, pero se demostró que alta ventilación es posible aun con poco viento. La ventilación natural perjudica la perdida de calor de los edificios, pero la carga mas fuerte de la tuberculosis se encuentra en climas calidos. Otros factores como tradiciones culturales o medidas de seguridad podrian resultar que las ventanas esten completamente cerradas durante la noche, pero este estudio ha demostrado que niveles de protección de ventilación se puede lograr con ventanas solo parcialmente abiertas. Ademas, los pabellones son menos hacinados durante la noche, y tambien podría ser posible usar controles ambientales adicionales, como la luz ultravioleta en la parte superirio de la sala. Aunque no sea apta para climas fríos, en países con clima tropical o subtropical posiblemente seria más seguro para los pacientes, trabajadores y visitas se abrigen bien en pabellones y salas de espera con ventanas grandes y abiertas con buena ventilación natural, para disminuir el riesgo de la transmisión de la tuberculosis intrahospitalaria. Mientras esta investigación ha enfocado en la TBC, la ventilación natural tambien tiene implicaciones para otras infecciones transmitidas por la via aerea, incluso la influenza, pero se debe notar que el efecto protector de ventilación disminuye cuando la infectividad sube[17].

Este estudio tiene varias limitaciones. El numero de salas con ventilación mecanica en el estudio (n=12) fue pequeño en comparación con el numero de salas con ventilación natural (n=70). Esto pudiera ha dejado una evaluacion injusto de ventilación mecanca en general, pero varias factores dicen que no. Primero, 9 de estas salas fueron salas de aislamiento respiratorio individual, y con un volumen promedio de 31 m^3^ son tipicos en tamaño. La proporción alta de salas individuales en la categoría de ventilación mecanica resultó en una tendencia de area del piso por persona en salas de ventilación mecanica actualmente mas grande que ella en salas de ventilación natural (11 *vs*. 9.3 m^2^ por persona) pero esta diferencia no fue estadísticamente significativo. Este favoria vlores aumentados por ventilación total calculado, y entonces valores disminuidos por riesgo de transmisión de TBC. Ademas, en la ventilación mecanica se asumió contar con 12 recambios de aire por hora según pautas, y esta bien documentado en el mundo verdadero que muchos sistemas de ventilación mecanica actualmente funcionan con niveles bajos de los recomendados. Otra limitación del estudio es las limitaciones intrinsicos del modelo Wells-Riley de infección aerea, que asume, por ejemplo, condiciones en estado de equilibrio, e infeccion siendo un ‘one-hit’ proceso, y no tiene en cuenta otros factores como el hecho de que un susceptible ubicado mas cerca de una fuente infecciosa tiene mas probabilidad ser infectado que una persona susceptible ubicada mas lejos. Tambien el modelo no tiene en cuenta la deposicion de bacteria en los alveoli, o la desaparición de particulas del aire debido a aterrizar por el piso. Sin embargo, no se presentan las cifras para el riesgo de transmsion de TBC como riesgo actual, pero como cifras relativas, para facilitar la comparación de la proteccion debido a ventilación natural en las salas antiguas y modernas, comparados con la ventilación mecanica.

En resumen, la ventilación natural tiene varias ventajas sobre la ventilación mecánica en la lucha contra la transmisión institucional de infecciones por vias aereas, especialmente en países de escasos recursos. En el diseño de establecimientos de salud hay lecciones del pasado, y posiblemente sea mejor re-emplazar el hacinamiento y poca ventilación con los conocimientos arquitectónicos de nuestros abuelos. Ambientes de aislamiento con presion negativa con buen mantenimiento son el estadard óptimo para cuidar a pacientes con infecciones respiratorias. Sin embargo, son demasiado costosos para lugares de escasos recursos, y se restringen para zonas pequenas designados de alto-riesgo, sin pensar en areas importantes de transmisión como servicios de emergencia y salas de espera. Cuando las personas potencialmente infectantes y personas susceptibles deben compartir ambientes, y el uso universal de respiradores y ventilación mecánica con presión negativa con buen mantenimiento no esta fácilmente disponible, se deben disminuir el hacinamiento y abrir las ventanas para maximizar la ventilación natural y disminuir el riesgo de la transmisión de la tuberculosis intrahospitalaria.

**Acknowledgments:** The authors would like to thank the staff of the following participating Lima hospitals for their invaluable support in making this research possible: Hospital Nacional Dos de Mayo; Hospital Nacional Daniel Alcides Carrión, Lima; Hospital de Apoyo Maria Auxiliadora; Instituto de Medicina Tropical Alexander von Humboldt; Hospital Nacional Hipolito Unanue; Instituto de Salud del Niño; Hospital Arzobispo Loayza; and Hospital Sergio Bernales, Collique. We also thank Edward Nardell for reviewing an earlier draft of this manuscript.

**Author contributions.** All authors contributed to the design or data analysis of the study, the writing of the article, and approval of the final version to be published. ARE had full access to all the data in the study and had final responsibility for the decision to submit for publication and is the guarantor.

# References:

1. Corbett, E.L., Watt CJ, Walker N, Maher D, Williams BG et al. (2003) *The growing burden of tuberculosis: global trends and interactions with the HIV epidemic.* Arch Intern Med **163**(9): 1009-21.

2. Valway, S.E., Greifinger RB, Papania M, Kilburn JO, Woodley C et al. (1994) *Multidrug-resistant tuberculosis in the New York State prison system, 1990-1991.* J Infect Dis **170**(1): 151-6.

3. Mohle-Boetani, J.C., Miguelino V, Dewsnup DH, Desmond E, Horowitz E et al. (2002) *Tuberculosis outbreak in a housing unit for human immunodeficiency virus-infected patients in a correctional facility: transmission risk factors and effective outbreak control.* Clin Infect Dis **34**(5): 668-76.

4. Dwyer, B., Jackson K, Raios K, Sievers A, Wilshire E et al. (1993) *DNA restriction fragment analysis to define an extended cluster of tuberculosis in homeless men and their associates.* J Infect Dis **167**(2): 490-4.

5. Curtis, A.B., Ridzon R, Novick LF, Driscoll J, Blair D et al. (2000) *Analysis of Mycobacterium tuberculosis transmission patterns in a homeless shelter outbreak.* Int J Tuberc Lung Dis **4**(4): 308-13.

6. Danis, K., Fitzgerald M, Connell J, Conlon M, Murphy PG. (2004) *Lessons from a pre-season influenza outbreak in a day school.* Commun Dis Public Health **7**(3): 179-83.

7. Ehrenkranz, N.J. and J.L. Kicklighter. (1972) *Tuberculosis outbreak in a general hospital: evidence for airborne spread of infection.* Ann Intern Med **77**(3): 377-82.

8. Petrosillo, N., E. Nicastri, and P. Viale. (2005) *Nosocomial pulmonary infections in HIV-positive patients.* Curr Opin Pulm Med **11**(3): 231-5.

9. Edlin, B.R., Tokars JI, Grieco MH, Crawford JT, Williams J et al. (1992) *An outbreak of multidrug-resistant tuberculosis among hospitalized patients with the acquired immunodeficiency syndrome.* N Engl J Med **326**(23): 1514-21.

10. Ikeda, R.M., Birkhead GS, DiFerdinando GT, Jr., Bornstein DL, Dooley SW et al. (1995) *Nosocomial tuberculosis: an outbreak of a strain resistant to seven drugs.* Infect Control Hosp Epidemiol **16**(3): 152-9.

11. Jiamjarasrangsi, W., N. Hirunsuthikul, and P. Kamolratanakul. (2005) *Tuberculosis among health care workers at King Chulalongkorn Memorial Hospital, 1988-2002.* Int J Tuberc Lung Dis **9**(11): 1253-8.

12. Jensen, P.A., Lambert LA, Iademarco MF, Ridzon R. (2005) *Guidelines for preventing the transmission of Mycobacterium tuberculosis in health-care settings, 2005.* MMWR Recomm Rep **54**(17): 1-141.

13. Beggs, C.B., Noakes CJ, Sleigh PA, Fletcher LA, Siddiqi K. (2003) *The transmission of tuberculosis in confined spaces: an analytical review of alternative epidemiological models.* Int J Tuberc Lung Dis **7**(11): 1015-26.

14. Riley, R.L. and E.A. Nardell. (1989) *Clearing the air. The theory and application of ultraviolet air disinfection.* Am Rev Respir Dis. **139**(5): 1286-94.

15. Granich, R., Binkin N J, Jarvis W R, Simone P M. (1999) *Guidelines for the prevention of tuberculosis in health care facilities in resource-limited settings*. WHO/CDS/TB99.269 ed. 1999, Geneva, Switzerland: World Health Organization.

16. Menzies, R., Schwartzman K, Loo V, Pasztor J. (1995) *Measuring ventilation of patient care areas in hospitals. Description of a new protocol.* Am J Respir Crit Care Med. **152**(6 Pt 1): 1992-9.

17. Nardell, E.A., Keegan J, Cheney SA, Etkind SC. (1991) *Airborne infection. Theoretical limits of protection achievable by building ventilation.* Am Rev Respir Dis **144**(2): 302-6.

18. Wells, W.F. (1955) *Airborne contagion and air hygiene*. Cambridge, MA: Harvard University Press.

19. Kennedy, W.J. and Bancroft, T.A. (1971) *Model-building for prediction in regression based on repeated significance tests.* Annals of Mathematical Statistics **42**: 1273-84.

20. Zeger, S.L., K.Y. Liang, and P.S. Albert. (1988) *Models for longitudinal data: a generalized estimating equation approach.* Biometrics **44**(4): 1049-60.

21. Zheng, B. (2000) *Summarizing the goodness of fit of generalized linear models for longitudinal data.* Statistics in Medicine **19**(10): 1265-1275.

22. SPSS, *SPSS User Manual Version 10.0*. 1999, Chicago: SPSS Inc.

23. Menzies, D., Fanning A, Yuan L, FitzGerald JM. (2000) *Hospital ventilation and risk for tuberculous infection in canadian health care workers. Canadian Collaborative Group in Nosocomial Transmission of TB.* Ann Intern Med **133**(10): 779-89.

24. Fraser, V.J., Johnson K, Primack J, Jones M, Medoff G et al. (1993) *Evaluation of rooms with negative pressure ventilation used for respiratory isolation in seven midwestern hospitals.* Infect Control Hosp Epidemiol **14**(11): 623-8.

25. Pavelchak, N., DePersis RP, London M, Stricof R, Oxtoby M et al. (2000) *Identification of factors that disrupt negative air pressurization of respiratory isolation rooms.* Infect Control Hosp Epidemiol **21**(3): 191-5.

26. Catanzaro, A. (1982) *Nosocomial tuberculosis.* Am Rev Respir Dis **125**(5): 559-62.

27. Pearson, M.L., Jereb JA, Frieden TR, Crawford JT, Davis BJ et al. (1992) *Nosocomial transmission of multidrug-resistant Mycobacterium tuberculosis. A risk to patients and health care workers.* Ann Intern Med **117**(3): 191-6.

28. Beck-Sague, C., Dooley SW, Hutton MD, Otten J, Breeden A et al. (1992) *Hospital outbreak of multidrug-resistant Mycobacterium tuberculosis infections. Factors in transmission to staff and HIV-infected patients.* JAMA **268**(10): 1280-6.

29. Coffey, C.C., Lawrence RB, Campbell DL, Zhuang Z, Calvert CA et al. (2004) *Fitting characteristics of eighteen N95 filtering-facepiece respirators.* J Occup Environ Hyg **1**(4): 262-71.

30. Biscotto, C.R., Pedroso ER, Starling CE, Roth VR. (2005) *Evaluation of N95 respirator use as a tuberculosis control measure in a resource-limited setting.* Int J Tuberc Lung Dis **9**(5): 545-9.

31. Bonifacio, N., Saito M, Gilman RH, Leung F, Cordova Chavez N et al. (2002) *High risk for tuberculosis in hospital physicians, Peru.* Emerg Infect Dis **8**(7): 747-8.

32. Riley, R.L., Mills CC, O'Grady F, Sultan LU, Wittstadt F et al. (1962) *Infectiousness of air from a tuberculosis ward. Ultraviolet irradiation of infected air: comparative infectiousness of different patients.* Am Rev Respir Dis **85**: 511-25.

33. Menzies, D. (1997) *Effect of treatment on contagiousness of patients with active pulmonary tuberculosis.* Infect Control Hosp Epidemiol **18**(8): 582-6.

34. Long, R., Zielinski M, Kunimoto D, Manfreda J (2002) *The emergency department is a determinant point of contact of tuberculosis patients prior to diagnosis.* Int J Tuberc Lung Dis, **6**(4): 332-9.

**Leyendas para figuras:**

# Figura 1: Método de medición de ventilación.

Ejemplo de un experimento de caída en la concentración de gas carbónico (C0_2_), que demuestra un rápido incremento al abrir el cilindro de gas hasta un pico de 6000 partes por. millón (ppm). Después se ve una caída lenta, que corresponde a 0.5 recambios de aire por hora (RAH), hasta que se abrieron las ventanas. Al abrir las ventanas, había una caída rápida en la concentración de C0_2_ que corresponde a 12 RAH. Experimentos repetidos de este tipo definieron los efectos de las variables arquitectónicas y ambientales sobre la ventilación

# Figure 2: Efecto de abrir ventanas y efecto de la velocidad del viento en la ventilación absoluta.

Se muestra el efecto de abrir algunas o todas de las ventanas y puertas, y la velocidad del viento sobre la ventilación natural, en comparación con salas de aislamiento respiratorio con ventilación mecánica y presión negativa. El trío de barras de la izquierda muestra la ventilación natural medida en días con poco viento (el quartil mas bajo, ≤2 km/h), con todas las puertas/ventanas cerradas (n=102), parcialmente abiertas (n=167) o todas abiertas (n=86). El trío de barras de la derecha representan ventilación absoluta los días con mas viento (los demás 3 quartiles combinados, i.e. >2 km/h) con todas las puertas/ventanas cerradas (n=266), parcialmente abiertas (n=74) o todas abiertas (n=240). ‘Parcialmente abierta’ fue definida como a lo menos una ventana o puerta abierta, pero no todas. La barra sola de la derecha representa la ventilación absoluta en salas de presión negativa con ventilación mecánica con 12 recambios de aire por hora. Los valores de recambios de aire por hora correspondientes a las 7 barras desde la izquierda hacia la derecha son: 1.0; 7.6; 20; 1.8; 17; 34; y 12.

**Figura 3: Ventilación y protección contra la transmisión de la tuberculosis en salas antiguas (construidas antes de 1950) y modernas (1970-90).**

Se muestra ventilación y protección contra la transmisión de la tuberculosis por el aire en la época antes de 1950 *vs.* las salas modernas con ventilación natural *vs* las salas de aislamiento respiratorio con ventilación mecánica y presión negativa. El trío de barras de la izquierda representa recambios de aire por hora en salas antiguas con ventilación natural (n=22; 201 experimentos), *vs*. salas modernas con ventilación natural (n=42; 125 experimentos), *vs*. salas de aislamiento con ventilación mecánica y presión negativa (n=12). El trío céntrico-izquierda representa la misma comparación pero por ventilación absoluta (m^3^/h/100); el trío céntrico-derecha representa lo de ventilación absoluta por persona (m^3^/h/100); y el trío de barras de la derecha representa el riesgo de transmisión de la tuberculosis por una exposición de 24 horas a pacientes sin tratamiento[17]. Se ve datos para las 64 salas con ventilación natural con ventanas y puertas todas abiertas (las 6 salas restantes no se podían abrir todas las ventanas).

**Figura 4: Riesgo estimado de la transmisión de tuberculosis por el aire por tres fuentes de infecioisdad diferentes, en salas antiguas con ventilación natural *vs*. salas modernas con ventilación natural *vs*. ventilación mecánica**

Riesgo de infección de tuberculosis con tiempo por exposición a casos de fuente de diferente infectividad en salas antiguas (pre-1950) con ventilación natural (lineas ‘dotted’) *vs*. salas modernas (1970-90) con ventilación natural (lineas ‘dashed’) *vs*. salas de aislamiento respiratorio con ventilación mecánica y presión negativa (año 2000)(lineas continuas). Las tres fuentes infecciosas son: q=1.3 pacientes de un pabellón de pacientes normales con tuberculosis[32](las tres lineas mas a bajos); q=13 un caso sin tratamiento que infectó a 27 colegas en su oficina durante 4 semanas[17](las tres lineas en el medio); q=60 un paciente con tuberculosis laringea [14](las tres lineas superiores). Valores medianos para todas las mediciones de ventilación absoluta por persona han sido utilizados en el modelo matematico por cada categoría de sala.

**Tabla 1:**

Un resumen de resultados son presentados para ventilación total (m^3^/h) medida en salas con ventilación natural con puertas y ventanas: cerradas; parcialmente abiertas; o todas abiertas; según velocidad del viento (quartil mas bajo, i.e. <2 km/h *vs*. la combinación de los tres quartiles demas i.e >2 km/h). ‘n’ significa el numero de experimentos. Todos los valores para ventilación mecanica han sido calculado asumiendo la ventilación de 12 recambios de aire por hora según pautas[12]. Estos datos se presentan gráficamente en figura 2. Medios y deviaciones estandares son medios geometricos y deviaciones estandartes geometricos porque los datos no fueron distribuidos normalemente. IQR = rango Inter.-quartile. SD = deviacion estándar.

| Configuración de ventanas y puertas | Ventilacion natural | | | | | | | | | | Ventilación mecánica | | | |
| --- | --- | --- | --- | --- | --- | --- | --- | --- | --- | --- | --- | --- | --- | --- |
|  | Viento <2 km/h | | | | | Viento >2 km/h | | | | |  |  |  |  |
|  | *n* | Mediana | IQR | Medio | SD | *n* | Mediana | IQR | Medio | SD | Mediana | IQR | Medio | SD |
| Todo cerrado | 102 | 91 | 40–205 | 87 | 3.7 | 266 | 136 | 59–307 | 133 | 3.3 | 402 | 330–1,209 | 520 | 1.9 |
| Parcialmente abierto | 167 | 473 | 240–833 | 460 | 2.7 | 74 | 1,780 | 988–3,042 | 1,650 | 2.1 |  |  |  |  |
| Todo abierto | 86 | 1,561 | 906–3,349 | 1,635 | 2.2 | 240 | 2,757 | 1,412–4,934 | 2,509 | 21 |  |  |  |  |

**Tabla 2**

Resumen de resultados por mediciones de ventilación y riesgo teorico de transmisión de TBC son presentados. Todas las mediciones por ventilación natural son con puertas y ventanas todas abiertas. Todos los valores para ventilación mecanica han sido calculados asumiendo una ventilación de 12 recambios de aire por hora según pautas[12].Riesgo de transmisión de TBC fue calculado usando el modelo de infeccion áerea Wells-Riley por exposición a pacientes tuberculosos generando 13 quanta infecciosa por hora (ver texto) [14, 17]. Estos datos se presentan gráficamente en la figura 3. Medios (mean significa medio, verdad?) y deviaciones estandares son medios geometricos y deviaciones estandartes geometricos porque los datos no fueron distribuidos normalemente. IQR = rango Inter.-quartile. SD = deviacion estándar.

| Tipo de ventilación | Recambios de aire por hora (hora^-1^) | | | | Ventilación total (m^3^/h) | | | | Ventilación total por persona (m^3^/h) | | | | Riesgo de transmission de TB (%) Transmission (%) | | | |
| --- | --- | --- | --- | --- | --- | --- | --- | --- | --- | --- | --- | --- | --- | --- | --- | --- |
|  | Mediana | IQR | Medio | SD | Mediana | IQR | Medio | SD | Mediana | IQR | Medio | SD | Median | IQR | Mean | SD |
| Toda ventilación natural | 28 | 18–46 | 28 | 4.7 | 2,477 | 1,162–4,345 | 2,241 | 5.4 | 1,053 | 516–1,749 | 942 | 4.8 | 16 | 10–30 | 17 | 2.8 |
| Ventilación natural construido pre-1950 | 40 | 26–52 | 38 | 5.0 | 3,769 | 2,477–5,104 | 3,401 | 6.1 | 1,557 | 1,063–2,283 | 1,508 | 5.3 | 11 | 7.9–16 | 12 | 3.2 |
| Ventilación natural construido 1970–1990 | 17 | 12–23 | 17 | 4.1 | 1,174 | 812–1,627 | 1,146 | 4.3 | 461 | 296–697 | 442 | 4.2 | 33 | 24–47 | 33 | 2.0 |
| Ventilación mecanica | 12 | — | 12 | — | 402 | 330–1,209 | 520 | 1.8 | 374 | 324–404 | 356 | 1.2 | 39 | 37–44 | 41 | 1.1 |

**Tabla 3: Determinantes de la ventilación natural y protección contra la transmisión de tuberculosis por el aire.**

Las variables ambientales y arquitectónicas que acercaron una asociación significativa con medidas de ventilación natural y riesgo de transmisión de tuberculosis en 70 salas con ventanas y puertas parcialmente o completamente abiertas (p<0.15) fueron incluidos en un modelo regresión multiple. Datos fueron normalizados por transformación logarítmica (base 10) para poder hacer analisis de regresión linear. CI = intervalos de confianza

| Determinante de ventilación | Recambios de aire por hora (log_10_) | | Ventilacion absoluta (m^3^/h) (log_10_) | | Riesgo de transmisión de la TB (log_10_) | |
| --- | --- | --- | --- | --- | --- | --- |
|  | Coefficiente (95% CI) | *p*-Valor | Coefficiente(95% CI) | p-Valor | Coefficient (95% CI) | *p*-Value |
| Area de puertas y ventanas abiertas (m^2^) | 0.027 (0.022 to 0.032) | <0.001 | 0.026 (0.022 to 0.031) | <0.001 | –0.024 (–0.027 to -0.020) | <0.001 |
| Presencia de puertas/ventanas abiertas por paredes opuestas | 0.337 (0.228–0.447) | <0.001 | 0.347 (0.235 to 0.460) | <0.001 | –0.216 (–0.290 to -0.142) | <0.001 |
| Altura del techo (m) | 0.064 (–0.002 to 0.130) | 0.056 | 0.108 (0.017 to 0.200) | 0.02 | –0.14 (–0.20 to -0.076) | <0.001 |
| Area del piso (m^2^) | –0.005 (–0.006 to - 0.004) | <0.001 | 0.005 (0.002 to 0.008) | <0.001 | 0.006 (0.004 to 0.007) | <0.001 |
| Velocidad del viento (km/h) | 0.034 (0.019 to 0.049) | <0.001 | 0.032 (0.017 to 0.048) | <0.001 | –0.028 (–0.040 to -0.016) | <0.001 |
| Altura sobre la tierra (m) | 0.004 (–0.003 to 0.010) | 0.2 | 0.006 (–0.000 to 0.013) | 0.06 | 0.002 (–0.003 to -0.007) | 0.4 |
| Constante | 0.599 (0.364 to 0.835) | <0.001 | 2.032 (1.747 to 2.317) | <0.001 | 2.172 (1.935 to 2.410) | <0.001 |
| Marginal R-square [21] | 0.635 | — | 0.675 | — | 0.663 | — |

**Figura 1**

**Figura 2**

**Figura 3**

**Figura 4**
